# Semi-Continuous Heterophase Polymerization to Synthesize Poly(methacrylic acid)-Based Nanocomposites for Drug Delivery

**DOI:** 10.3390/polym14061195

**Published:** 2022-03-16

**Authors:** Hugo A. Andrade-Melecio, Víctor H. Antolín-Cerón, Abraham G. Alvarado-Mendoza, Milton Vázquez-Lepe, Karla A. Barrera-Rivera, Antonio Martínez-Richa, Sergio M. Nuño-Donlucas

**Affiliations:** 1 Departamento de Ingeniería Química, Centro Universitario de Ciencias Exactas e Ingenierías, Universidad de Guadalajara, Guadalajara 44430, Mexico; hugo_andrade07@hotmail.com; 2 Departamento de Ciencias Básicas Aplicadas e Ingeniería, Centro Universitario de Tonalá, Universidad de Guadalajara, Tonalá 45425, Mexico; vhaceron@hotmail.com; 3 Departamento de Química, Centro Universitario de Ciencias Exactas e Ingenierías, Universidad de Guadalajara, Guadalajara 44430, Mexico; gabriel.alvarado@academicos.udg.mx; 4 Departamento de Ingeniería de Proyectos, Centro Universitario de Ciencias Exactas e Ingenierías, Universidad de Guadalajara, Guadalajara 44430, Mexico; milton.vazquez@academicos.udg.mx; 5 Departamento de Química, Universidad de Guanajuato, Guanajuato 36050, Mexico; fionita@ugto.mx (K.A.B.-R.); richa28@msn.com (A.M.-R.)

**Keywords:** carbon nanotubes, nanocomposites, semi-continuous heterophase polymerization, poly(amidoamine) dendrimer, methacrylic acid, nano-carrier, hydrocortisone

## Abstract

The design of nanocomposites with the potential for drug delivery is a topic of great interest. In this work, the synthesis of nanocomposites of poly(methacrylic acid) (PMAA) grafted onto carbon nanotubes (CNTs) functionalized with poly(amidoamine) (PAMAM) dendrimer by semicontinuous heterophase polymerization *SHP*, at three different methacrylic acid (MAA) dosing rates, is reported. *SHP* is a polymerization technique poorly used to prepare nanocomposites containing CNTs and has the potential to produce more ordered alkyl methacrylic polymer chains, which could favor the obtaining of a homogenous nanocomposite. For the nanocomposites synthesized, a lowest addition rate monomer-starved condition was reached. Analysis by X-ray photoelectron spectroscopy (XPS), and thermogravimetric analysis (TGA) demonstrate that functionalized CNTs are grafted onto the PMAA matrix. The ability of prepared nanocomposites to deliver hydrocortisone was evaluated by ultraviolet-visible spectroscopy (UV-Vis). The hydrocortisone release profiles of pure PMAA and of their nanocomposites prepared at the lowest monomer fed rate were fitted with Higuchi and Korsmeyer–Peppas models, successfully. Functionalized CNTs have a crucial role to induce an effective release of hydrocortisone from the prepared nanocomposites.

## 1. Introduction

Carbon nanotubes-based polymer nanocomposites are a noteworthy class of composites with a high potential to be used in diverse fields [1,2]. Typically, polymer nanocomposites (PNs) filled with carbon nanotubes (CNTs) are classified as structural or functional composites in function of their specific applications [3]. For functional composites, relevant properties of CNTs, such as its magnetic, thermic, or optical properties, among others, are of interest. As another type of carbon material, which has extensive applications in sensing, energy conversion and storage, and organic catalysis [4], functional composites are widely demanded at the technology level, for example, for developing energy storage devices [5] or medical smart materials [6], among others [7].

In the field of PNs focused on biomedical applications, there is a current interest in the designing of new drug nano-carriers with the effective ability to release drugs. To achieve this goal, a series of challenges in the design of these materials must be overcome. Specifically, to induce targeted drug delivery, it is necessary to increase nano-carrier efficacy. In this sense, preparation of stimuli-responsive nano-carriers are of special interest because they can carry antitumor drugs to specific sites and exert control when drug releasing, thus limiting the chance of side effects [8].

Poly(methacrylic acid) is a biodegradable, ionizable hydrophilic, pH-sensitive polymer [9,10]. The ionization and deionization behavior of their carboxylic acid groups confers to PMAA a high sensitivity to pH changes in the environment. PMAA remains at a coiled shape at pH values less than five, because its –COOH groups are not ionized. Comparatively, at high pH values, PMAA chains acquire an expanded conformation, due to the fact that –COOH functional groups are ionized. This leads to repulsion between –COOH ionized groups [10]. For this, PNs prepared using PMAA as a polymeric matrix can perform as a pH-sensitive nano-carrier in aqueous medium. This fact improves the potential interest in prepared PNs because the use of water-dispersed pH-sensitive sensors are of current interest, as was reported elsewhere [11].

The use of CNTs for biomedical applications is a topic which is not exempt from controversy. Contradictory reports about CNTs toxicity have been published. On the one hand, Liu et al., reported that pristine or oxidized CNTs can enter cells without producing toxic effects [12], which would allow their use to deliver drugs in targeting sites. Moreover, the internal cavity of CNTs can be used as drug reservoirs [13], and in consequence, CNTs could be used as drug delivery vehicles [14]. However, on the other hand, several reports reveal potential dangerous health side effects associated to CNTs exposure. In this sense, based on studies on animals, evidence was found of damage induced by CNTs on several type of soft tissues. In addition, it is noteworthy to mention that the exact cause of CNTs toxicity is not yet known, completely [15]. Nevertheless, there are several reports indicating that functionalized CNTs have lesser toxic effects in respect to unfunctionalized CNTs [16,17,18]. Moreover, Wick et al., reported that CNTs’ toxicity diminishes in a proportional way with the augment in solubility degree or dispersion [19]. Similarly, Sadegh et al. documented that adequate dispersed CNTs have no toxicity or induce little damage both in vitro and in vivo [20]. As a consequence, achieving high dispersion of CNTs induced by their functionalization in a polymeric matrix makes possible the use of these outstanding nanomaterials in nanomedicine. The chemical functionalization of CNTs and of carbon-based materials is a difficult task. Moreover, after being achieved, interesting materials are produced, for example: (i) obtaining of CNTs with tunable chemical groups on their surface available to meet a specific aim [21], (ii) preparation of graphene quantum dots from oxidization of multi-walled carbon nanotubes (MWCNTs) [22] or, (iii) synthesis of a sensor from covalent functionalization of carbon nanoparticles [23]. To create polymer brushes covalently grafted onto CNTs, three main approaches have been applied: (i) the “grafting to” approach, which consists of the attachment of polymer chains (typically commercial polymers) onto the surface of previously functionalized CNTs [24], (ii) the “grafting from” approach, which is a strategy whereby the polymerization of monomers from a surface-derived initiator that was previously attached covalently to the surface of CNTs is carried out [25], and (iii) the “grafting through” strategy, which involves the CNTs’ surface becoming functionalized with a polymerizable group [26]. All mentioned strategies have the goal of achieving success through CNTs’ surface modification because this improves dispersion of CNTs into the polymer matrix of a nanocomposite [27].

Dendrimers are a structured polymer candidate for using in biomedical applications, such as drug delivery [28]. PAMAM dendrimers have a nanoscopic spherical structure, which is formed by amidoamine units that contain repeating dendritic branching [29]. The use of PAMAM dendrimer to prepare PNs has been reported previously [30]. Functionalization of CNTs with poly(amidoamine) (PAMAM) dendrimer improves CNTs’ reactivity [31]. For this way, PNs where CNTs’ surface has plenty of amino groups can be produced. The size and structure of PAMAM dendrimer is sensitive to the pH environment, which favors its utilization as drug delivery vehicles at desirable pH (typically in a range of pH from 5 to 7.4) [32]. Moreover, the globular shape of the PAMAM dendrimer [33] can help to prepare homogeneous CNTs-based PNs, as the physical shape of the PAMAM dendrimer is crucial to inducing exfoliation between different carbon nanotubes. Because achieving a high dispersion of CNTs in a polymeric matrix is a difficult task [34], attaching PAMAM dendrimer onto functionalized CNTs could be an interesting first step to preparing PNs with highly dispersed CNTs.

Semicontinuous heterophase polymerization (*SHP*) is a relatively novel polymerization technique, in which a neat monomer is added semi-continuously at a controlled feeding rate to a reaction flask, which contains a monomer-free aqueous solution of surfactant and initiator [35]. The aim of using a controlled feed rate of the monomer is to achieve a monomer-starved condition. Using *SHP* can help to prepare PNs that contain CNTs to be chemically attached to an acrylate monomer [36]. Moreover, it has been shown that suitable polymers can be prepared to deliver drugs by *SHP* [37]. It is notable to mention that due to alkyl methacrylate type polymers, prepared by *SHP* under monomer-starved condition can develop tacticity [38], it could be possible that this technique would induce favorable conditions to synthesize more ordered polymer chains, which favors a homogeneous CNTs dispersion into a methacrylic polymer used as a polymer matrix of a PN.

The aim of this work is to synthesize a PN of PMAA grafted onto CNTs functionalized with PAMAM dendrimer by *SHP*, and evaluate their capacity to deliver hydrocortisone (11,17,21-trihydroxypregn-4-ene-3,20-dione). Because hydrocortisone is a drug used to treat several types of cancer [39], the preparation of CNTs-based PNs with a polymer matrix that is pH-sensitive, via an experimental technique which could reduce the CNTs’ self-agglomeration and, in consequence, could slow down their toxicity, is of actual interest.

## 2. Experimental Section

### 2.1. Materials

Methacrylic acid (MAA) (99%), sodium dodecylsulfate (SDS) (98%), dichloromethane (ACS reagent), oxalyl chloride (OxCl) (98%), triethylamine (Et_3_N) (99%), methyl acrylate (MA) (99%), *N,N*-dimethylformamide (DMF) (99.8%), ethylene diamine (EDA) (>99%), and toluene (ACS reagent) were purchased from Sigma-Aldrich (St. Louis, MO, USA). Potassium persulfate (KPS) (ACS reagent) and nitric acid (70%) were acquired from Fermont, Mexico. Absolute alcohol (99.5%), Fe(NO_3_)_3_·9H_2_O (98.2%) and hydrochloric acid (ACS reagent) were provided from Golden Bell (Guadalajara, Mexico). Alumina boats were acquired from Alfa Aeser (Tewksbury, MA, USA). Gas nitrogen (99.99%) was purchased from INFRA (Guadalajara, Mexico), and argon (99.998%) was provided from PRAXAIR (Guadalajara, Mexico). Deionized and bi-distilled water was purchased from Productos Selectropura (Guadalajara, Mexico). All mentioned chemical reagents were used without further purification.

### 2.2. Synthesis and Purification of Synthesized CNTs

CNTs were synthesized by chemical vapor deposition (CVD) technique following an experimental procedure that was used previously by our group, and published elsewhere [40]. CNTs were purified using the following procedure: 0.5 g of CNTs were added to 100 mL of a mixture of concentrated H_2_SO_4_ and HNO_3_ (3:1 *v*/*v*), placed in a round-bottom flask. The mixture was stirred for 2 h at 30 °C. After that, the product was filtered and the filtrate was washed out several times with bi-distilled water until achieving a pH = 7. Then, washed CNTs were dried at 50 °C in a vacuum oven until constant weight. The purified CNTs were named CNTs_pur_.

### 2.3. Functionalization of CNTs

**Scheme 1:**
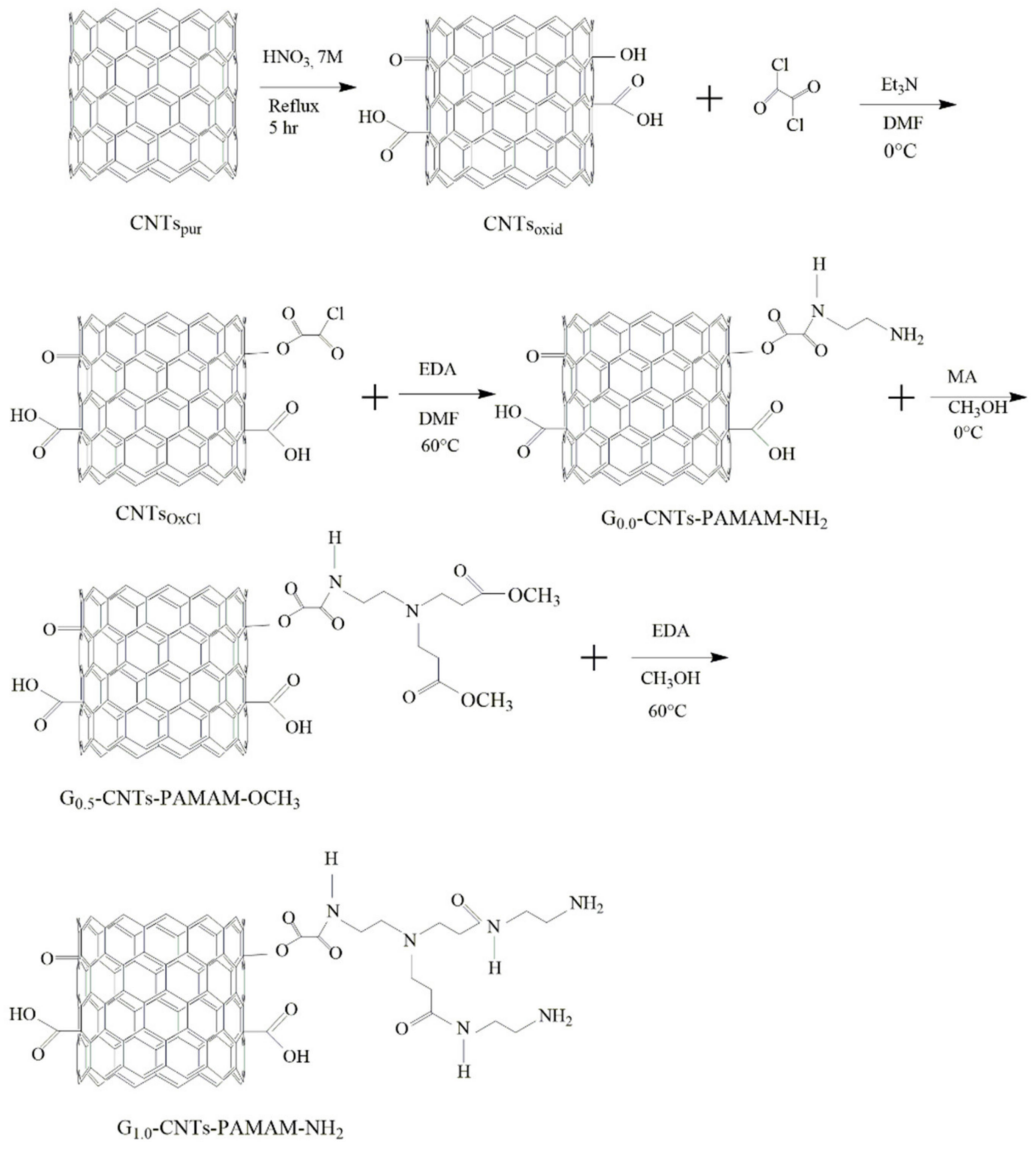
Chemical route used to prepare G1.0-CNTs-PAMAM-NH_2_.

CNTs_pur_ were chemically functionalized with the purpose to attach poly(amidoamine) dendrimers with amine ending groups onto their walls. For this, we implement a light modification of a procedure published previously elsewhere [41]. The procedure followed by us has several steps. As an initial condition necessary to achieve the success of the CNTs’ functionalization developed in this work, it is necessary to obtain CNTs with hydroxyl and carboxyl groups attached to their surface, and so in an initial step, CNTspur were treated with an acid solution. First, 1 g of CNTs_pur_ was introduced into a 250 mL glass Soxhlet apparatus. Afterward, 100 mL of a nitric acid aqueous solution 7 M was added. The mixture was refluxed at least for 5 h under the local atmospheric pressure (0.85 bar). The product is a partial oxidized CNTs and was named CNTs_oxid_. CNTs_oxid_ were washed with bi-distilled water several times until the elimination of nitric acid residues. After this, they were dried in an oven at 50 °C for 24 h. Through this treatment, hydroxyl, carboxyl, and carbonyl groups were created on the CNTsoxid’s surface The content of carboxyl and hydroxyl groups was determined through indirect titration procedures, as it was reported previously elsewhere [36,42]. Second, 1.5 g of completely dried CNTs_oxid_ were placed into a 250 mL glass reactor and 100 mL of DMF was added. The mixture was sonicated for 20 min. After this, the reactor was immersed into an ice-water bath at 0 °C and 10 mL of Et_3_N was added. After this, 2 mL of OxCl was added drop by drop. The mixture was allowed to react for 2 h under constant stirring with N_2_ bubbling. Afterwards, the mixture was allowed to reach room temperature. After this, the mixture was immersed into an oil bath at 60 °C for 2 h to evaporate the residual OxCl. Then, it was centrifuged and filtered. The filtrate was washed several times with DMF. The last wash was performed with THF. The solid product, named CNTs_OxCl_, was dried in a vacuum oven at 60 °C for 48 h. Third, 15 mL of EDA were mixed with 40 mL of DMF. To this mixture was added 3 g of CNTs_OxCl_. The resulting mixture was sonicated for 15 min and it was heated at 60 °C for 48 h. The product was allowed to cool to room temperature. After this, it was centrifuged at 4200 rpm for 5 min. Then it was filtered, and the filtrate was washed 3 times with DMF. Finally, it was dried in a vacuum oven at 50 °C until achieving constant weight. The product was the generation zero (G_0.0_) of the amino-functionalized CNTs and was named G_0.0_-CNTs-PAMAM-NH_2_. Fourth, G_0.0_-CNTs-PAMAM-NH_2_ were modified following the aza-Michel addition of methyl acrylate to increase amino end groups. For this, 3 g of G_0.0_-CNTs-PAMAM-NH_2_ was placed into a glass reactor of 250 mL. Then, 50 mL of methanol was added. The mixture was sonicated for 15 min. Afterwards, the mixture was immersed in ice-water bath at 0 °C. A total of 6 mL of methyl acrylate were added dropwise under constant stirring. The mixture was kept at 0 °C for 2 h. After this time, the reactor was removed from the ice-water bath and left to reach room temperature. Then, it was immersed into an oil bath at 60 °C for 24 h to eliminate the methanol. The product contains methoxy terminal groups and was named G_0.5_-CNTs-PAMAM-OCH_3_. Fifth, to recreate the terminal amine groups, 3 g of G_0.5_-CNTs-PAMAM-OCH_3_ were placed in glass reactor of 250 mL. Then, 50 mL of methanol were added. The mixture was sonicated for 15 min. The reactor was immersed into the oil bath at 60 °C. A total of 15 mL of EDA were added drop by drop. The mixture was allowed to react for 24 h under constant stirring and with nitrogen bubbling. Then, the product obtained was centrifuged at 5000 rpm for 10 min. The precipitated was dried at 50 °C until constant weight was obtained. This product was named first-generation poly(amidoamine) attached to functionalized CNTs (G_1.0_-CNTs-PAMAM-NH_2_) dendrimer. The fourth and the fifth steps were repeated twice. The last product, named G_3.0_-CNTs-PAMAM-NH_2_, was dried in a vacuum oven at 50 °C until constant weight. Finally, it was stored in a desiccator until later use. The G_3.0_-CNTs-PAMAM-NH_2_ were used to prepare the nanocomposites studied in this work. Scheme 1 presents the chemical route followed to obtain the G1.0-CNTs-PAMAM-NH_2_.

### 2.4. Synthesis of Poly(methacrylic acid) and Their Poly(MAA)–CNTs Nanocomposites by SHP

PMAA was synthesized by *SHP*. All reactions used the same content of SDS: 5 wt.%, and the initiator (KPS 1 wt.% respect to MAA). There were two different formulations prepared: (i) one used a content of 4 wt.% of MAA, while (ii) for the other, the content of MAA was 8 wt.%. On the one hand, during the synthesis carried out with 4 wt.% of MAA, initially, the adequate amount SDS (5 wt.%) was placed in a glass reactor of 250 mL. After that, 82 wt.% of water was added. The mixture was heated at 60 °C, keeping continuous stirring and under nitrogen bubbling. After 30 min, 1 wt.% of KPS respect to content of MAA dissolved in 0.96 wt.% of water was added. A mixture of 4 wt.% of MAA and 8 wt.% of water was introduced in a glass syringe (BD Yael, Franklin Lakes, NJ, USA) of 20 mL This syringe was placed in a dosing pomp (KD Scientific, Holliston, MA USA). The reaction started when the monomer feed commenced. After adding the complete amount of monomer, the mixture was allowed to react for one more hour. The complete content of water used in this formulation was 90.96 wt.% On the other hand, when 8 wt.% of MAA was used, a similar procedure was followed, but now after placed 5 wt.% of SDS in the glass reactor, 69.59 wt.% of water was added. Then, a solution of 1 wt.% of KPS dissolved in 1.33 wt.% of water was added. The two mixtures of 4 wt.% of MAA in 8 wt.% of water were introduced in two glass syringes. The content of the first syringe was dosed to the mixture placed into the glass reactor. When the dosage finished, immediately the content of the second syringe was dosed. Now, the total content of water was 86.92 wt.%. For the two formulations mentioned, three monomer-feeding rates (0.1, 0.2, or 0.3 g/min) were used.

The synthesis of the nanocomposites was carried out by a similar procedure using the same amount of SDS, MAA, and KPS. Again, two formulations based on content of 4 or 8 wt.% of MAA were used. When the synthesis was performed with 4 wt.% of MAA, prior to the synthesis of the nanocomposite, 2 wt.% of MAA was mixed with a certain amount of G_3.0_-CNTs-PAMAM-NH_2_. The exact mass of G_3.0_-CNTs-PAMAM-NH_2_ used corresponds to 1 or 0.5 wt.% with respect to the total mass of MAA used for this synthesis. The mixture obtained was sonicated for 15 min at room temperature. Then, 4 wt.% of water was added to this mixture. The new mixture was sonicated for other 15 min. After this, the last mixture was introduced into the glass syringe. The monomer feeding was performed through two shots. For the first shot, dissolution of 2 wt.% of MAA dissolved in 4 wt.% of water was introduced into a glass syringe. For the action of the pump, this monomer amount was introduced to glass reactor. Immediately after the first shot, a second charge (formed by the mixture of the 2 wt.% of MAA, 4 wt.% of water and, the adequate amount of G_3.0_-CNTs-PAMAM-NH_2_) was fed through the pump pulse. When the synthesis was carried out with 8 wt.% of MAA, the preparation followed the same procedure, but now, the first shot was a mixture of 4 wt.% of MAA in 8 wt.% of water. In addition, the second shot was a mixture of 4 wt.% of MAA, 8 wt.% of water, and the necessary amount of G_3.0_-CNTs-PAMAM-NH_2_. For both formulations, the same aforementioned monomer feed rates used to prepare pure PMAAs were used. The total amount of water used was adjusted to keep the same wt.% percentages of SDS, MAA, and KPS that were used for the synthesis of PMAA by *SHP*.

Table 1 lists the legends to identify the name of homopolymers of PMAA and of the nanocomposites prepared, as well as the specific content of G_3.0_-CNTs-PAMAM-NH_2_ employed, and the rate of addition used.

**Scheme 2:**
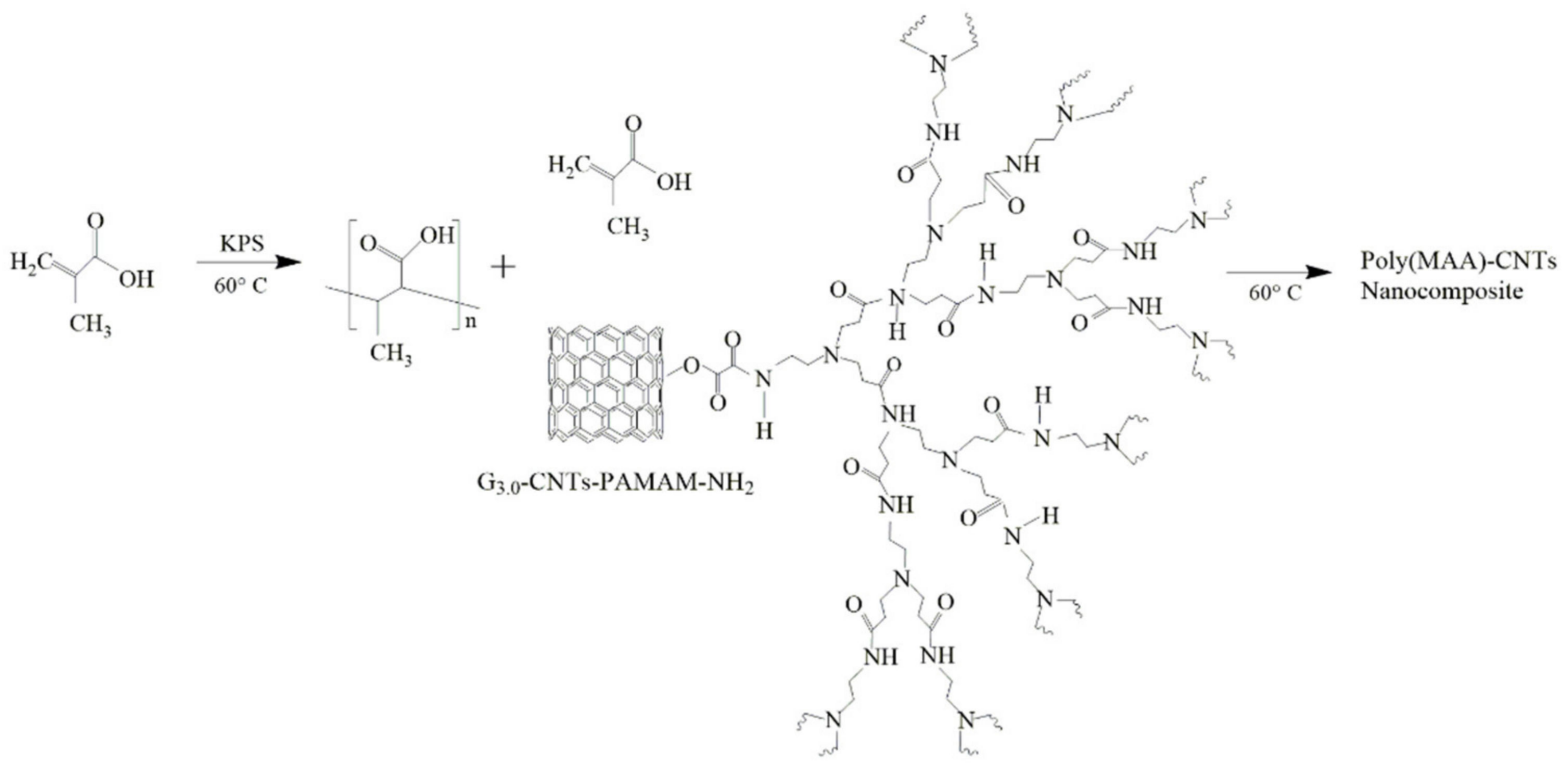
Chemical route followed to synthesize the poly(MAA)–CNTs nanocomposites.

In Scheme 2, the chemical route used to prepare the poly(MAA)–CNTs nanocomposites is shown.

### 2.5. Characterization Techniques

In order to determine conversion, samples of pure PMAAs or of their nanocomposites were withdrawn from the glass reactor at given times. Then, adding few drops of 0.05 M hydroquinone solution, the polymerization reaction was stopped. Global and instantaneous conversions were determined by gravimetry.

Previously to characterization of the PMAA and their nanocomposites by a battery of techniques, the latex obtained from each polymerization reaction was dialyzed for 1 week in a bath of bi-distilled water at 40 °C. Every day the water of the bath was changed. After this, water was removed in an oven at 40 °C until a solid of constant weight was obtained.

The analysis by Fourier transform infrared spectroscopy (FT-IR) of samples of synthesized and functionalized CNTs was carried in a spectrophotometer of the Perkin Elmer model Spectrum One. For this, dry samples of CNTs were mixed with dry KBr. After that, pellets were prepared by compression. Recorded spectra from 4000 to 450 cm^−1^ were obtained, taken from an average of 45 scans to reduce the signal/noise ratio, and at a resolution of 2 cm^−1^.

PMAA and their nanocomposites were analyzed by the X-ray photoelectron spectroscopy (XPS) technique. The system employed has an XR 50 M monochromatic Al Ka1 (hv = 1468.7 eV) X-ray source. In addition, it has a Phoibos 150 spectrometer with one-dimensional detector 1D-DLD, which was provided by SPECS (Berlin, Germany). Before analysis, samples were deposited on a steel sample holder through cooper tape to avoid interface from the carbon tape and were dried in a vacuum oven for 48 h. After this, samples were introduced into the pre-chamber. The base pressure used to make the measurements was 4.2 × 10^–10^ mbar. Measurements were recorded with an electron takeoff angle of 90° at 150 W, setting the pass energy at 10 eV and step size of 0.1 eV. A flood gun device was employed as a compensating charge on samples. All XPS spectra were shifted using the reference of C–C binding energy position.

Differential scanning calorimetry (DSC) was used to thermically characterize the PMAAs and their nanocomposites. DSC thermograms were recorded on a TA Instruments calorimeter model Q-100 (New Castle, DE, USA). To obtain the DSC thermograms, a dynamic heating program from 40 to 200 °C was followed at a heating rate of 10 °C/min. A flow rate of 50 mL/min of nitrogen was used to maintain an inert atmosphere in the sample cell of the DSC equipment. There were two scans obtained and the second scan is reported.

Thermogravimetric analysis (TGA) of the nanocomposites and of pure PMAAs synthesized was obtained on a TA Instruments thermobalance model TGA5000 Discovery. For this, the sample masses used were in the range of 3 to 10 mg. TG curves were recorded by heating the samples from 50 to 600 °C at 10 °C/min under a nitrogen atmosphere created by flow rate of 25 mL/min.

UV-VIS spectroscopy was utilized to determine the hydrocortisone in vitro release from tablets of PMAAs and of their nanocomposites. To make this, tablets of the nanocomposites or their pure polymeric matrix were prepared. Tablets were made by mixing a certain amount of mentioned materials (in the range of 35 to 55 mg) with 1% (*w*/*w*) of hydrocortisone at room temperature. The mixture was placed in a 7 mm diameter mold. Then it was pressed to obtain a tablet with 1.5 mm of thickness. After this, the tablet was placed in 3 mL of potassium biphthalate buffer at pH 5 in a quartz cell. The evolution of hydrocortisone release was studied by determination of hydrocortisone concentration evaluating the absorbance as a function of time. For this, a Perkin Elmer spectrometer Lambda 25 was used at 248 nm.

## 3. Results and Discussion

### 3.1. Characterization by FT-IR Spectroscopy of the Functionalized Carbon Nanotubes

**Figure 1:**
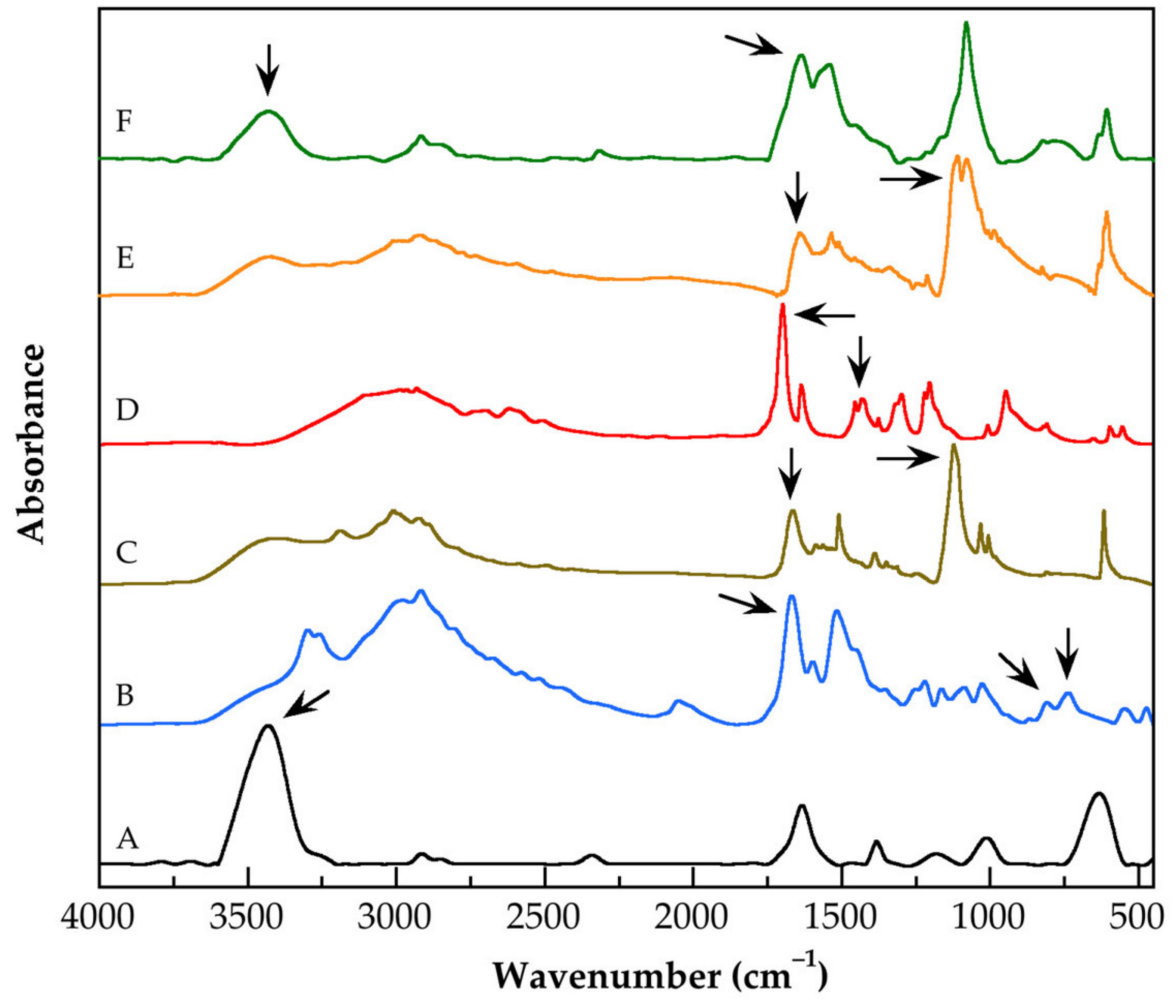
FT-IR spectra of CNTs_oxid_ (**A**), CNTs_OxCl_ (**B**), G_0.0_-CNTs-PAMAM-NH_2_ (**C**), G_0.5_-CNTs-PAMAM-OCH_3_ (**D**) G_3.0_-CNTs-PAMAM-NH_2_ (**E**) and a mixture of G3.0-CNTs-PAMAM-NH_2_ and MAA (**F**).

For the versatile fields in which FT-IR technique can be applied, because of their sensitivity, simplicity, and easy calibration, FT-IR is a highly appreciated technique [43]. Between their fields of application, their capacity to analyze nanomaterials as the nanocomposites is noteworthy [44]. In this respect, the purpose of analyzing nanomaterials by FT-IR spectroscopy is to detect their functional groups; in the case of surface-modified nanomaterials, this is particularly useful in order to verify if a particular chemical route of modification was carried out successfully. Figure 1 depicts FT-IR spectra of the purified and functionalized CNTs, as well as of a mixture of G_3.0_-CNTs-PAMAM-NH_2_ with MAA. The most relevant bands of each spectrum are described below. Some of these are indicated with arrows to clarify the chemical route followed to functionalize each type of CNTs, which is shown in Scheme 1. Table 2 lists the marked bands, the chemical group whose vibration cause the indicated band, and the corresponding wavelength. In the spectrum of the CNTs_oxid_ (Figure 1A), at 3433 cm^−1^ the band, due to stretching vibration of the hydroxyl groups, was detected. At 1634 cm^−1^ the band originated by stretching vibration of C=C bond conjugated to carbonyl group was observed. As a shoulder of this band, a weak band at 1710 cm^−1^ caused by stretching vibration of free carboxyl groups attached to the walls of the CNTs was identified. In the spectrum of the CNTs_OxCl_ (Figure 1B) the band at 1668 cm^−1^ was assigned to the stretching vibration of the di-carbonyl functionality (–O–(C=O)–(C=O)–) attached to carbon aromatic rings of the CNTs’ walls. This assignment is based in a previous report, which states that the band caused by stretching vibration of di-carbonyl groups is detected usually near 1670 cm^−1^ [45]. There were two bands detected at 808 and 737 cm^−1^, due to the stretching vibration of the C–Cl bond. In addition, at 1517 cm^−1^ the harmonic of this stretching vibration appears. The spectral behavior described confirms that acyl chloride groups of OxCl reacted with hydroxyl groups of CNTs_oxid_, creating di-carbonyl functionality and, in the CNTs_OxCl_ surface, there are acyl chloride groups available to react in a later step. As expected, the spectra of G_0.0_-CNTs-PAMAM-NH_2_ (Figure 1C) and G_3.0_-CNTs-PAMAM-NH_2_ (Figure 1E) have a similar pattern, with little differences. Thus, in the spectrum of the G_0.0_-CNTs-PAMAM-NH_2_, the band (named amide I) of the stretching vibration of the C=O bond included in the (–(C=O)–NH–R–) functionality appears at 1663 cm^−1^; while in the spectrum of G_3.0_-CNTs-PAMAM-NH_2_, it was detected at lower frequencies (1641 cm^−1^). Similar behavior was observed to the band caused by stretching vibration of the N–H bond, which appears at 3410 cm^−1^ (G_0.0_-CNTs-PAMAM-NH_2_) and at 3427 cm^−1^ (G_3.0_-CNTs-PAMAM-NH_2_). Intense bands due to stretching vibration of the C–N bond of the C–N–C functionality are observed at 1123 cm^−1^ (G_0.0_-CNTs-PAMAM-NH_2_) and at 1112 cm^−1^ (G_3.0_-CNTs-PAMAM-NH_2_), but in the last case, it is resolved as a double band. These results confirm that PAMAM dendrimer chains were created from CNTs_OxCl_ walls. The FT-IR spectrum of the G_0.5_-CNTs-PAMAM-OCH_3_ (Figure 1D) shows clear differences with respect to the spectrum of G_0.0_-CNTs-PAMAM-NH_2_. Thus, at 1699 cm^−1^ a hydrogen-bonded carbonyl band was detected. These hydrogen bonds are dimers formed between N–H functionality and the C=O bond. The localization of mentioned spectral contribution has been documented in polymer blends [46], and in supramolecular complexes [47], among others. At higher frequencies, as a shoulder of the hydrogen-bonded carbonyl band, a weak band at 1717 cm^−1^ was detected. Stretching vibration of the free carbonyl group of ester functionality causes this band. These results show that into the G0.5-CNTs-PAMAM-OCH_3_ predominates the hydrogen bonds between N–H and C=O bonds. This fact indicates high inter-association between chains attached to CNTs because the amount of complementary groups is enough to bring them closer, until achieving hydrogen bonds formation. Finally, at 1432 cm^−1^ a band caused by symmetric deformation of the C–H bond of the –(C=O)–O–CH_3_ functionality was recorded. In order to promote competition between chemical groups that can interact by physical interactions (for example via hydrogen bonds), specifically between carboxyl groups of MAA and N–H and C=O of G_3.0_-CNTs-PAMAM-NH_2_, both components were mixed before the preparation of the nanocomposites. The FT-IR spectrum of a mixture of G3.0-CNTs-PAMAM-NH_2_ and MAA (Figure 1F) shows at 1637 cm^−1^ an intense spectral contribution typically named band amide II, which is caused by vibration bending of N–H bond of the NH_2_ functionality. As a shoulder of this band, at 1717 cm^−1^ the band appears due to stretching vibration of free carbonyl group. At 1540 cm^−1^ another intense band was detected, due to bending vibrations of N–H bonds not associated to hydrogen bonds. This means that inter-association between N–H and C=O bonds detected in the G_0.5_-CNTs-PAMAM-OCH_3_ and also in the G_3.0_-CNTs-PAMAM-NH_2_ are modified by the presence of carboxyl groups of MAA monomer. The existence of NH_2_ groups that do not participate in hydrogen bonding is confirmed by the band detected at 3431 cm^−1^, which is caused by stretching vibration of N–H bonds. This means that in the analyzed mixture, there are free NH_2_ groups available to react in a later step.

### 3.2. Kinetic of Polymerization of PMAA and of Their Nanocomposites Synthesized by SHP

**Figure 2:**
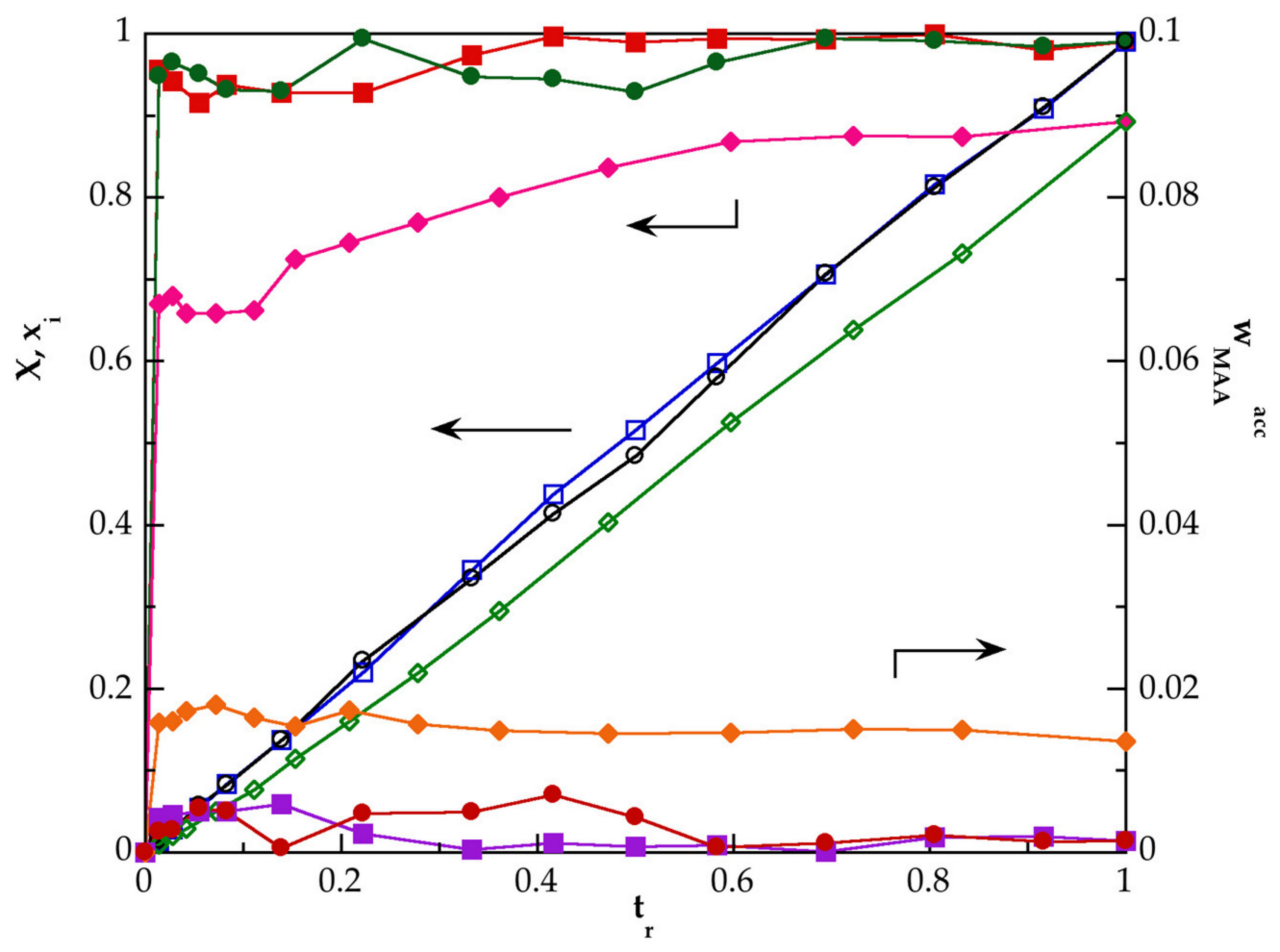
Advancement of instantaneous, *x_i_* (close symbols) and global *X* (open symbols) conversions and weight fraction of residual monomer, $w_{MAA}^{acc}$ (close symbols) with relative time for the polymerization of pure MAA to synthesize the PMAA 2 (♦ ◇) or for the polymerization of a mixture of MAA and G_3.0_-CNTs-PAMAM-NH_2_ to prepare the nanocomposites PMAA–CNTs 3 (■ ☐), and PMAA–CNTs 4 (● ◯) at a monomer feeding rate of 0.1 g/min.

Figure 2 shows the evolution of the instantaneous (*x_i_*) and the global (*X*) conversions, as well as the weight fraction of the residual monomer ($w_{MAA}^{acc}$) in function of the normalized time *t_r_*, which was calculated as the ratio of monomer addition time *t* divided by the total addition time, for the synthesis of PMAA 2, PMAA–CNTs 3, and PMAA–CNTs 4 at a monomer-feeding rate of 0.1 g/min. The use of the normalized time allows a practical comparison of the conversion data for different monomer-feeding rates. This is because the time of monomer fed change as a function of monomer content used for a particular reaction and the feeding rate used. Both the instantaneous and global conversions were calculated through gravimetric measurements of samples taken at specific times via mass balances and applying the following formulae:

(1) $$x_{i}\left(t\right)=\frac{w_{prod}}{tR_{a}}$$

(2) $$X\left(t\right)=\frac{t\cdot R_{a}\cdot x_{i}}{M_{total}}\;$$

where $w_{prod}$ is the amount of PMAA or PMAA-based nanocomposite produced (dependent on the type of synthesis performed) up to time *t*, $R_{a}$ is the monomer feeding rate, $M_{total}$ is the total weight of monomer added, and $w_{MAA}^{acc}\;$is the weight fraction of the residual monomer, which is calculated as the ratio of the weight of non-reacted monomer and the total of the reaction mixture at given time. The evolution of global conversion of the PMAA and of the nanocomposites PMAA–CNTs 3 and PMAA–CNTs 4 is nearly linear, but for the mentioned nanocomposites detected, values of *X(t)* close to 1 at *t_r_* = 1, for PMAA 2 it is 0.89 at the same *t_r_*. With respect to the instantaneous conversion of the PMAA–CNTs 3 and PMAA–CNTs 4, nanocomposites reach high values (0.96) at low value of normalized time (*t_r_* = 0.02), after this, there is a change in a few reaching values almost equal to 1 from *t_r_* = 0.41 for the PMAA–CNTs 3, and from *t_r_* = 0.70 for the PMAA–CNTs 4, keeping this *x_i_(t)* until the end of the polymerization reaction. For the PMAA 2, *x_i_(t)* increases constantly until *t_r_* = 0.59, then practically did not change (*x_i_(t)* = 0.86) and never reached values close to 1. The weight fraction of residual monomer of the mentioned nanocomposites is very small and close to zero for both nanocomposites from *t_r_* = 0.58. This fact suggests strongly that the monomer-starved condition was achieved for the synthesis of the nanocomposites. However, for the polymerization of PMAA 2, it is not possible to ensure that the monomer-starved condition has been reached, because the values of $w_{MAA}^{acc}\;$remain practically constant during all times of the polymerization reaction and were lowered to 0.02.

**Figure 3:**
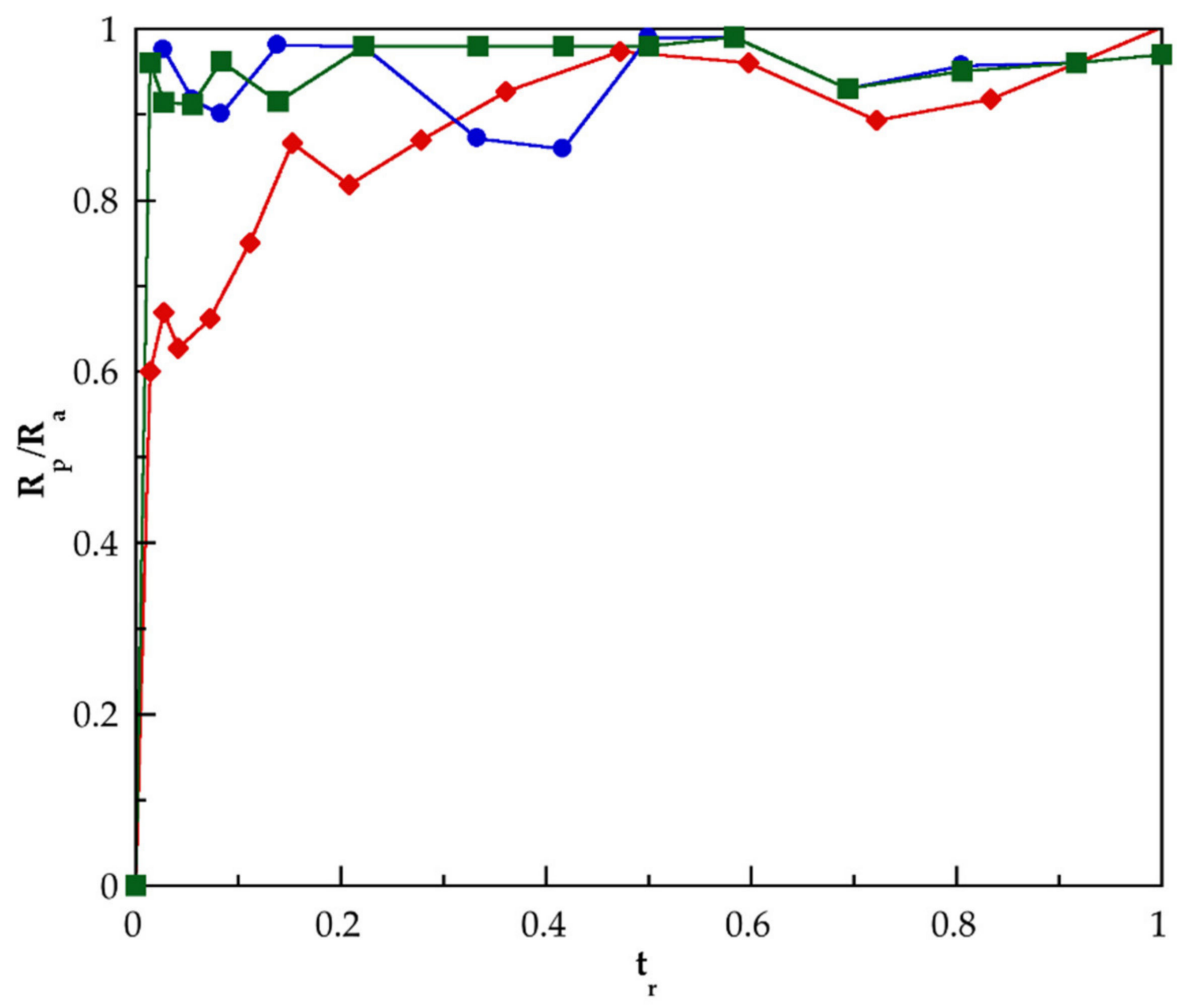
Evolution of the rate of polymerization to monomer feeding rate (*R_p_/R_a_*) in function of normalized time for polymerization of MAA to prepare the PMAA 2 (♦) or for the polymerization of a mixture of MAA and G_3.0_-CNTs-PAMAM-NH_2_ to obtain the nanocomposites PMAA–CNTs 3 (■) and PMAA–CNTs 4 (●) at a monomer feeding rate of 0.1 g/min.

Figure 3 shows the progress of the ratio of the rate of polymerization to monomer-feeding rate (*R_p_/R_a_*) in function of normalized time for polymerization of MAA to prepare the PMAA 2, and the nanocomposites PMAA–CNTs 3 and PMAA–CNTs 4. For the nanocomposites, this ratio rises and reaches values close to 1 at *t_r_* = 0.02 and remains at high values practically during all reaction time. This fact confirms that the polymerization reaction of mentioned nanocomposites was carried out under a monomer-starved condition [36,38,47]. Comparatively, the evolution of the ratio of *R_p_/R_a_* in function of *t_r_* for PMAA 2 confirm that monomer-starved condition is not reached during the synthesis of this homopolymer. Similar results were observed for PMAA 1 preparation (obtained without reaching a monomer-starved condition) and for the synthesis of the nanocomposites PMAA–CNTs 1 and PMAA–CNTs 2 (prepared under monomer-starved condition). These last materials were also prepared at a monomer-feeding rate of 0.1 g/min.

**Figure 4:**
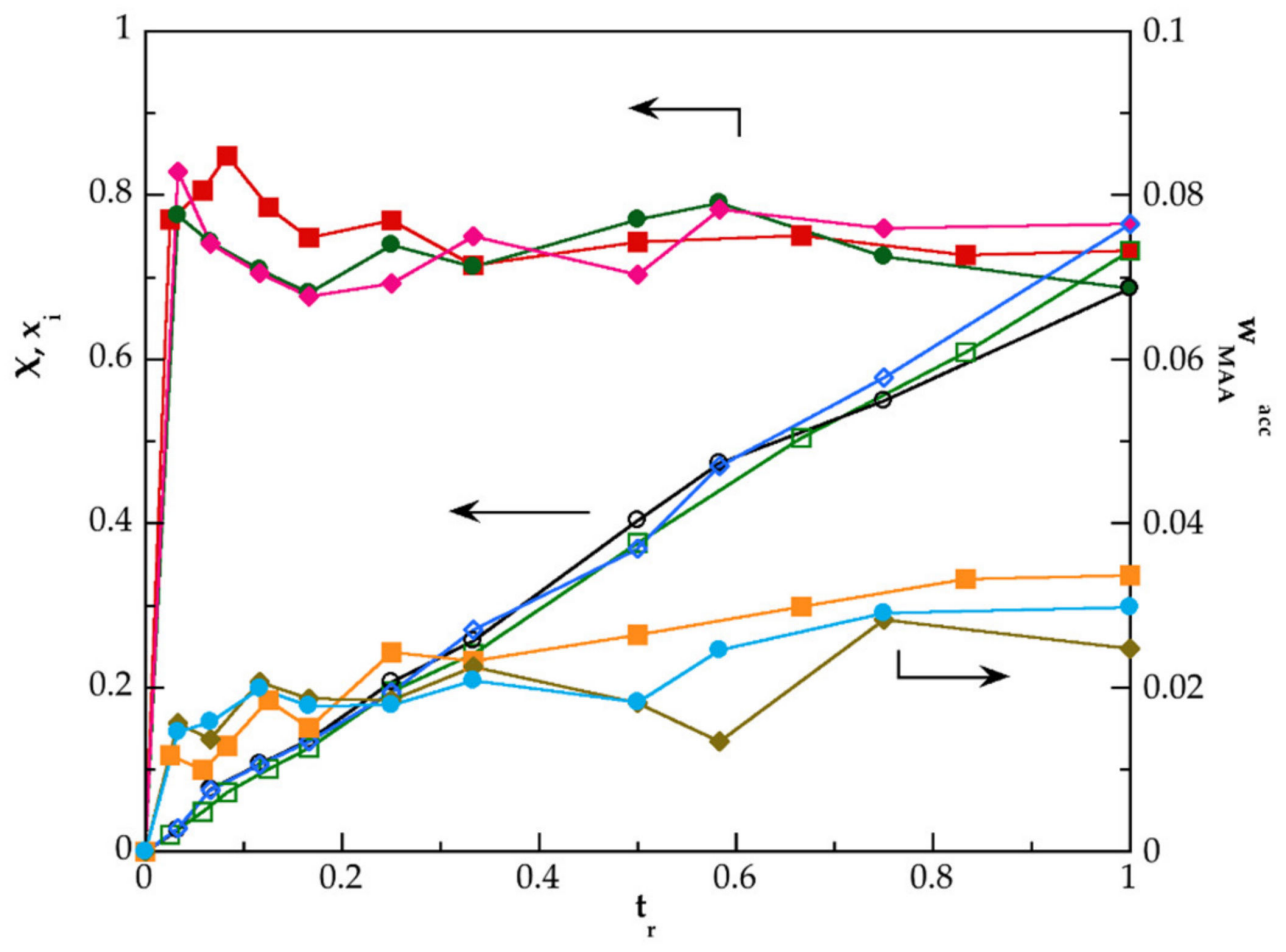
Progress of instantaneous *x_i_* (close symbols) and global *X* (open symbols) conversions and weight fraction of residual monomer, $w_{MAA}^{acc}$ (close symbols) with relative time for the polymerization of pure MAA to synthesize the PMAA 6 (■ ☐) or for the polymerization of a mixture of MAA and G_3.0_-CNTs-PAMAM-NH_2_ to prepare the nanocomposites PMAA–CNTs 11 (● ◯) and PMAA–CNTs 12 (♦ ◇) at a monomer feeding rate of 0.3 g/min.

**Figure 5:**
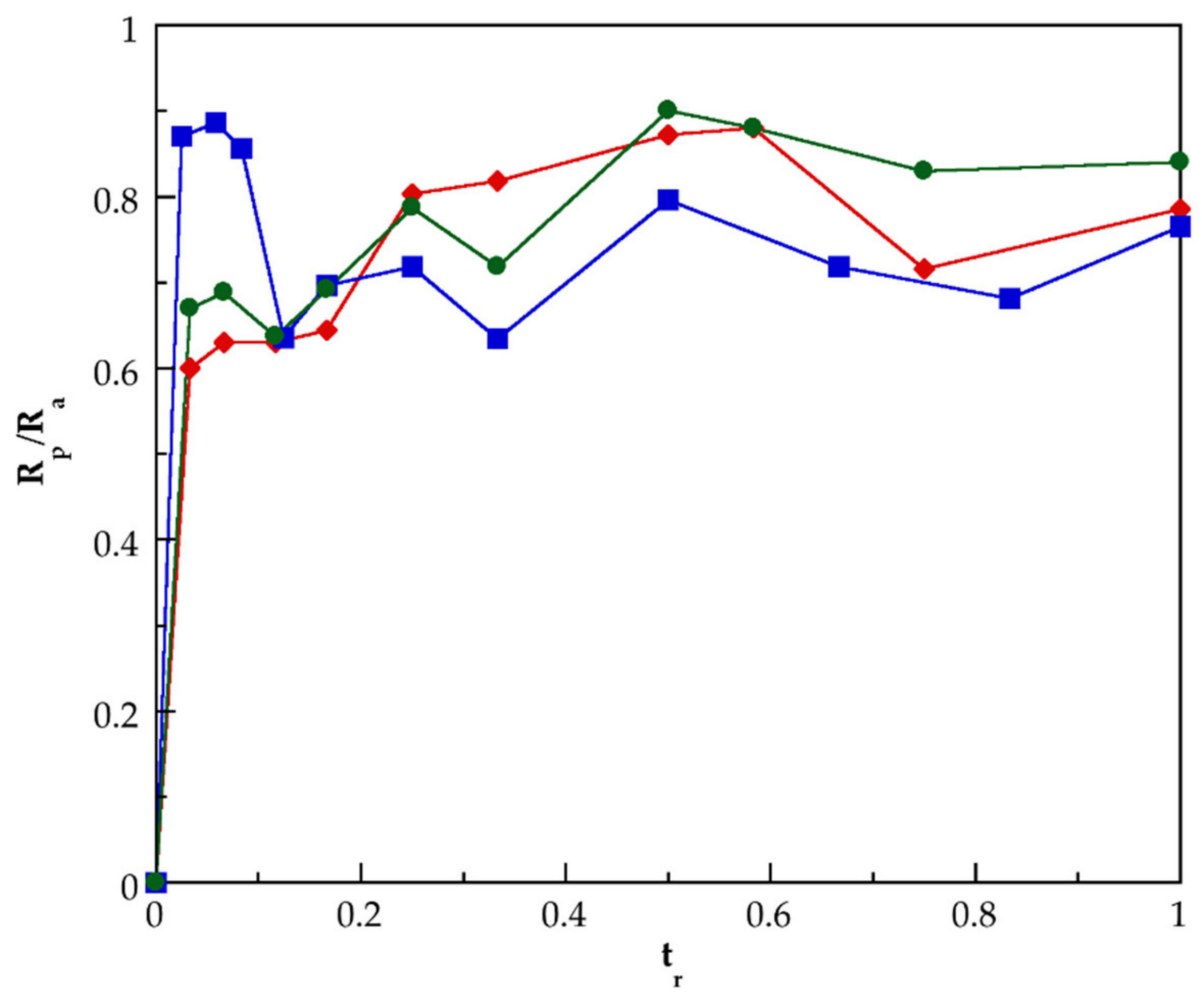
Development of the rate of polymerization to monomer feeding rate (*R_p_/R_a_*) in function of normalized time for polymerization of MAA to synthesize the PMAA 6 (■) or for the polymerization of a mixture of MAA and G_3.0_-CNTs-PAMAM-NH_2_ to prepare the nanocomposites PMAA–CNTs 11 (●) and PMAA–CNTs 12 (♦) at a monomer feeding rate of 0.3 g/min.

When the monomer-feeding rate increases, the reaction kinetics changes. Figure 4 presents the progress of *x_i_*, *X,* and $w_{MAA}^{acc}$ as *t_r_* increases for PMAA 6 and the nanocomposites PMAA–CNTs 11 and PMAA–CNTs 12 synthesized at a monomer-feeding rate of 0.3 g/min, while Figure 5 shows the advancement of the ratio of *R_p_/R_a_* in function of *t_r_*, also for these same materials. As can be observed, for none of these materials did the polymerization develop under the monomer-starved condition. In a similar fashion, for PMAA 5 and the nanocomposites PMAA–CNTs 9 and PMAA–CNTs 10, the monomer-starved condition was not reached.

At an intermediate monomer feeding rate (0.2 g/min), contradictory results were found, while PMAA–CNTs 5 and PMAA–CNTs 6 did not reach monomer-starved condition, and for PMAA–CNTs 7 and PMAA–CNTs 8, it seemed that the mentioned condition was reached in certain periods of time. As would be expected for the homopolymers PMAA 3 and PMAA 4, the monomer-starved condition was not reached during any time.

These results imply that the CNTs play a crucial role in the polymerizations in which monomer-starved condition was reached. The observed behavior can be explained by taking into consideration that CNTs have free-radical scavenging capacity [48,49,50]. Both MWCNTs and SWCNTs have scavenging activity against free radicals. Fenoglio et al. reported that MWCNTs have a remarkable capacity to act as scavengers of hydroxyl or superoxide radicals [51], while Lucente-Schultz et al. proved that functionalized SWCNTs have a scavenging activity, which is very sensitive to their structural modifications [52]. Moreover, Amiri et al., confirmed that MWCNTs functionalized with amino acids present high antioxidant activity and can acts as excellent scavengers of free radicals [53]. Therefore, it is evident that the G_3.0_-CNTs-PAMAM-NH_2_ acts as an effective scavenger of free radicals on the studied polymerization systems. Then, primary radicals would create growing chains from CNTs walls, additionally to the ones that can grow from the –NH_2_ end groups of the dendrimers chains. The number of all growing chains must be enough to trap all monomer molecules until *R_a_* = *R_p_*. When the nanocomposites were prepared at a monomer-feeding rate of 0.1 g/min, the mentioned capacity of the functionalized CNTs was enough to induce monomer-starved condition, but as the monomer feed rate increased, the high amount of MAA molecules inhibited the functionalized CNTs’ ability to meet the monomer-starved condition, until this condition was lost, because not all monomer molecules reacted. When the polymerization was carried out without the presence of the G_3.0_-CNTs-PAMAM-NH_2_, it is reasonable to consider that there is a lower amount of growing PMAA chains with respect to the ones in the synthesis of the mentioned nanocomposites. Therefore, during any time, not all monomer molecules reacted on available growing chains and the monomer-starved condition was not reached.

### 3.3. Evaluation of the Grafting of Functionalized CNTs Onto the Polymeric Matrix of the Prepared Nanocomposites

**Figure 6:**
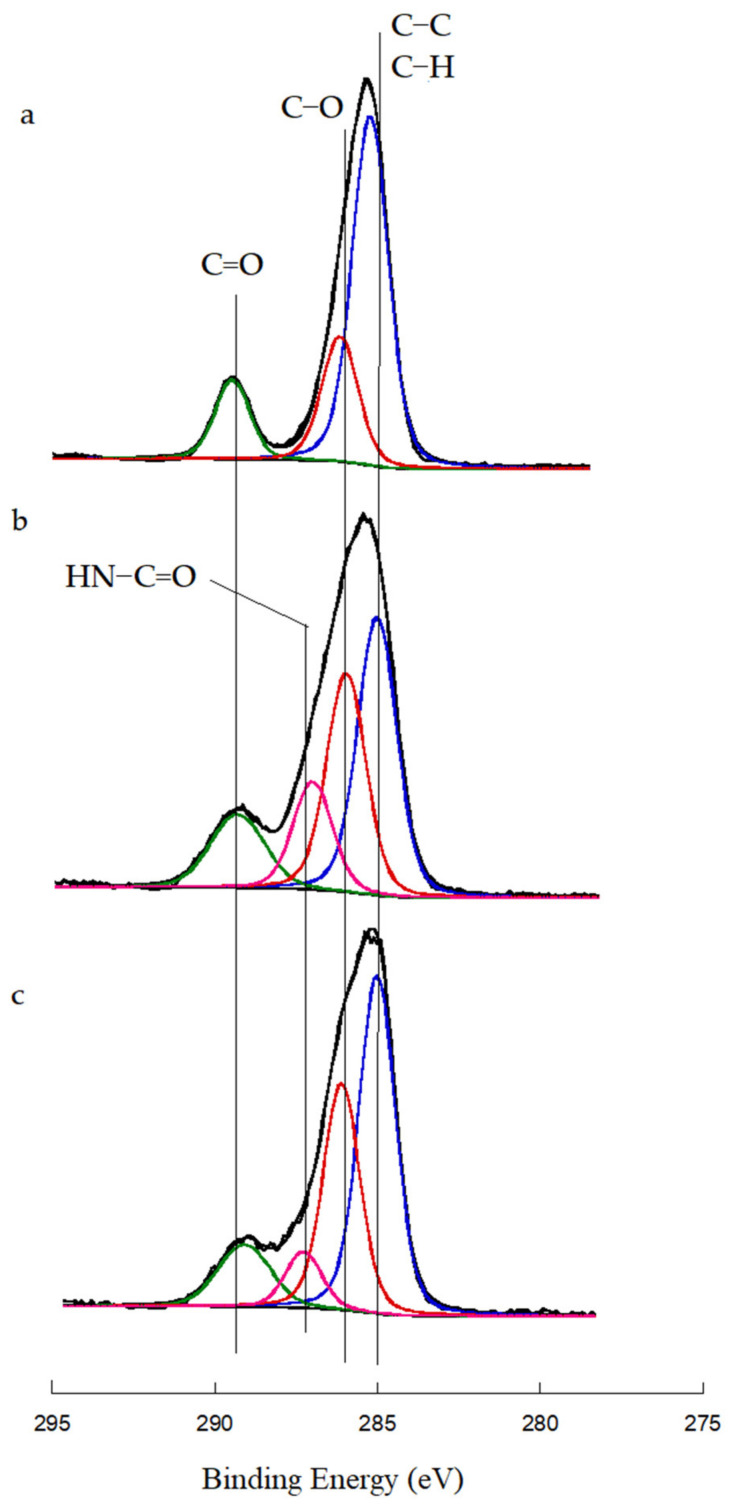
C*1s* core level normalized spectra of PMAA 2 (**a**), PMAA-G_3.0_-CNTs 3 (**b**), and PMAA-G_3.0_-CNTs 4 (**c**).

**Figure 7:**
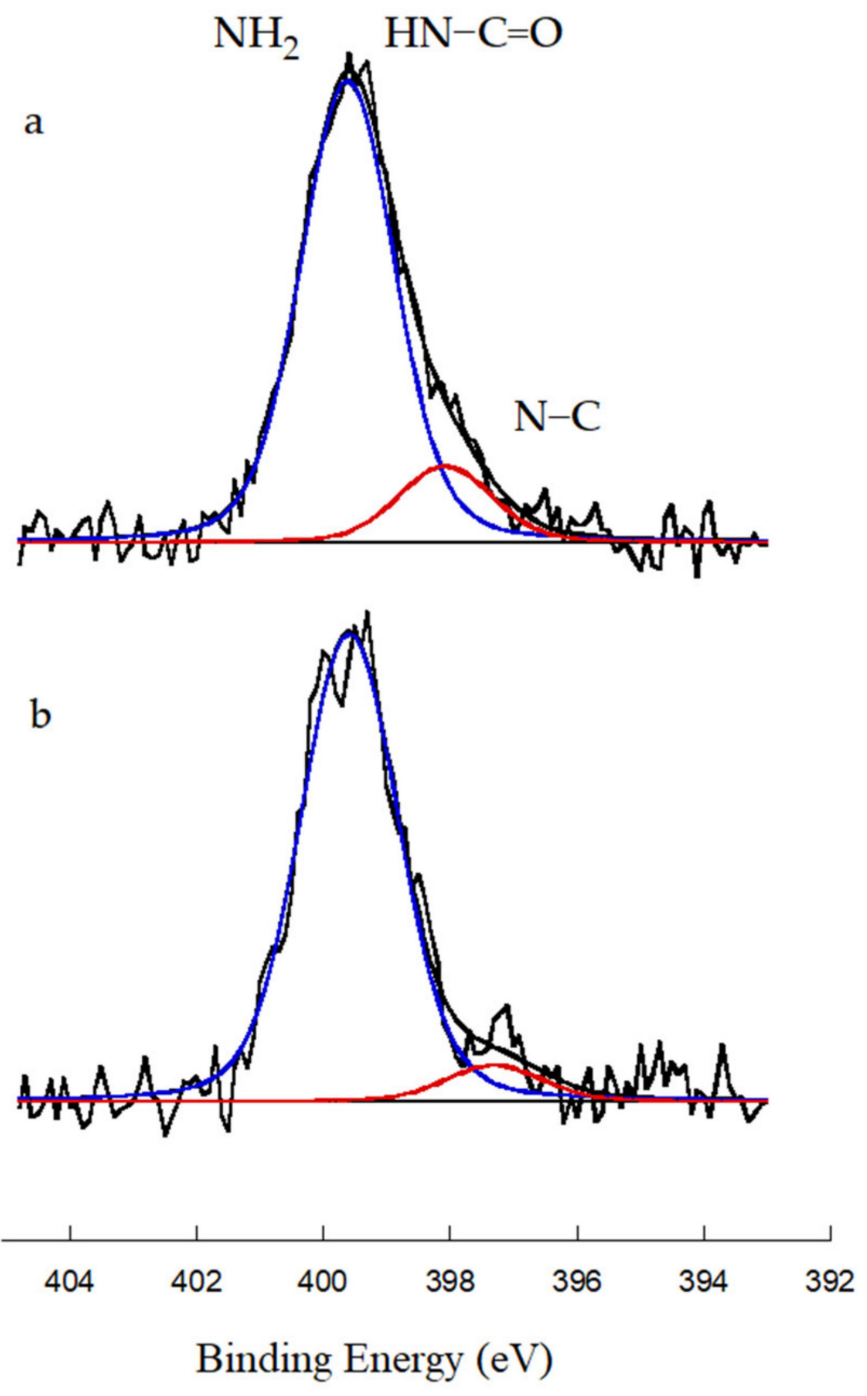
N1s core level normalized spectra of PMAA-G3.0-CNTs 3 (**a**) and PMAA-G3.0-CNTs 4 (**b**).

The electronic structure and the chemical composition of PMAA, PMAA-G3.0-CNTs 3, and PMAA-G3.0-CNTs 4 were also investigated by X-ray photoelectron spectroscopy (XPS), thus providing information on a possible grafting reaction between the functionalized CNTs and the polymer chains of PMAA. To assess the formation of covalent bonds in complex systems, XPS technique has demonstrated a high capacity, as has been published in previous works [54,55]. Figure 6 shows the C*1s* core level normalized spectra of PMAA 2, PMAA-G_3.0_-CNTs 3, and PMAA-G_3.0_-CNTs 4. The mentioned homopolymer and their nanocomposites were prepared at the same monomer feed rate (0.1 g min^−1^) and with the same content of MAA (8 wt.%). All data were peak fitted using the active background approach with the software Analyzer v 1.42 [56]. C–C bonding position at 284.8 eV was used to adjust each spectrum to the appropriate scale. The C*1s* core level normalized spectrum of PMAA 2 depicts at three positions caused by C–C/C–H bonds at 284.7 eV [57], C–O bond at 285.0 eV [53,54], and C=O functionality at 289.0 eV [57,58]. The calculated atomic concentration of these groups was 31.0%, 24.3%, and 10.0%, respectively. For the nanocomposites, the C*1s* core level normalized spectrum shows an additional component. This consists of a peak at 287.2 eV that was assigned to –OC–NH–bond [23,59,60]. Thus, in the C*1s* core level normalized spectrum of PMAA-G_3.0_-CNTs 3, the positions, components, and their atomic concentration were 284.7 eV (C–C, C–H) 20.4%, 285.0 eV (C–O) 16%, 287.2 eV (–OC–NH–) 7.9%, and 289.0 eV (C=O) 7.3%, respectively. In a similar fashion for PMAA-G_3.0_-CNTs 4, the positions, components, and their atomic concentration were 284.7 eV (C–C, C–H) 20.3%, 285.0 eV (C–O) 13.9%, 287.2 eV (–OC–NH–) 5.4%, and 289.0 eV (C=O) 3.5%, respectively. Figure 7 shows the N1s core level normalized spectra of PMAA-G3.0-CNTs 3 and PMAA-G3.0-CNTs 4. In both spectra, two signals were detected. In the N1s core level normalized spectrum of PMAA-G3.0-CNTs 3, the signals were detected at 399.6 eV and at 397.6 eV, the first was assigned to NH_2_/–HN–C=O groups [60,61] and their atomic concentration was 3.8%, while the second signal was assigned at N–C bonds [62,63,64] and their atomic concentration was 0.65%. For PMAA-G3.0-CNTs 4, the first signal, also assigned to NH_2_/–HN–C=O groups, was detected at 399.6 eV and their atomic concentration was 3.0%, while the second signal was detected at 398 eV and corresponds to C–N bonds, and their atomic concentration was 0.24%.

**Scheme 3:**
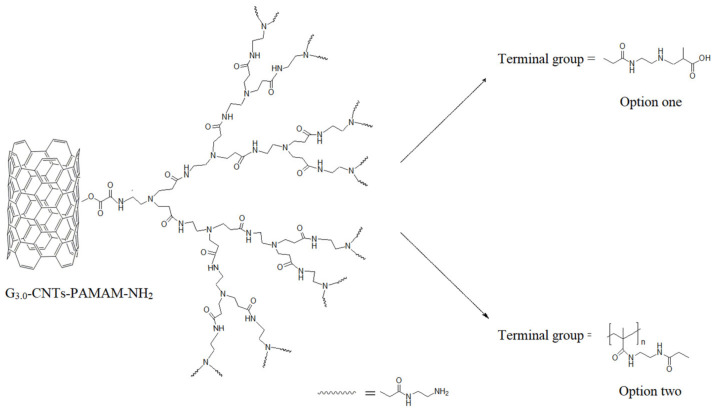
Chemical structure of the poly(MAA)–CNTs nanocomposite with the terminal groups of the two grafting reactions: (**i**) aza-Michael reaction (option one) or, (**ii**) amidation reaction (option two).

In the studied system, there are two possible ways by which –NH_2_ functionality can react: (i) by the well-know amidation reaction, where carboxyl groups of MAA or PMAA react through a thermal activation, or (ii) by the aza-Michael reaction, where the amine group is added to carbon–carbon double bond of an unsaturated compound [65], for example with C=C bond presents in the MAA monomer. PMAA chains, created in the first shot of the *SHP* process used to prepare the mentioned nanocomposites, have one carboxyl group for each monomeric unit. This fact suggests that there are high possibilities that these carboxyl groups react. However, it is necessary to overcome the high activation energy barrier, and keep high temperatures (typically higher than 140 °C) in the system, so that the two necessary conditions to produce amidation reaction are activated thermally with good yields, which lower the success of mentioned reaction under the experimental route used. On the contrary, aza-Michael reaction is a common reaction that occurs at room temperature, without catalyst, and mostly in neat conditions. The Spartan 14 Wavefunction software (taking advantage of 3–21 G Hartree–Fock model) was used to calculate the free energy of the amidation reaction and of the aza-Michael reaction at 95 °C, resulting in −38 kJ/mol and −74 kJ/mol, respectively. Thus, although both reactions are favorable, under the mentioned calculus, aza-Michael reaction is the most probable to occur. The analyzed results indicate that the nanocomposites prepared consist of G_3.0_-CNTs-PAMAM-NH_2_ grafted to PMAA chains. In Scheme 3, the chemical structure of the poly(MAA)–CNTs nanocomposite with the terminal groups of the two grafting reactions: (i) aza-Michel reaction (route one) or, (ii) amidation reaction (route two), is presented.

**Figure 8:**
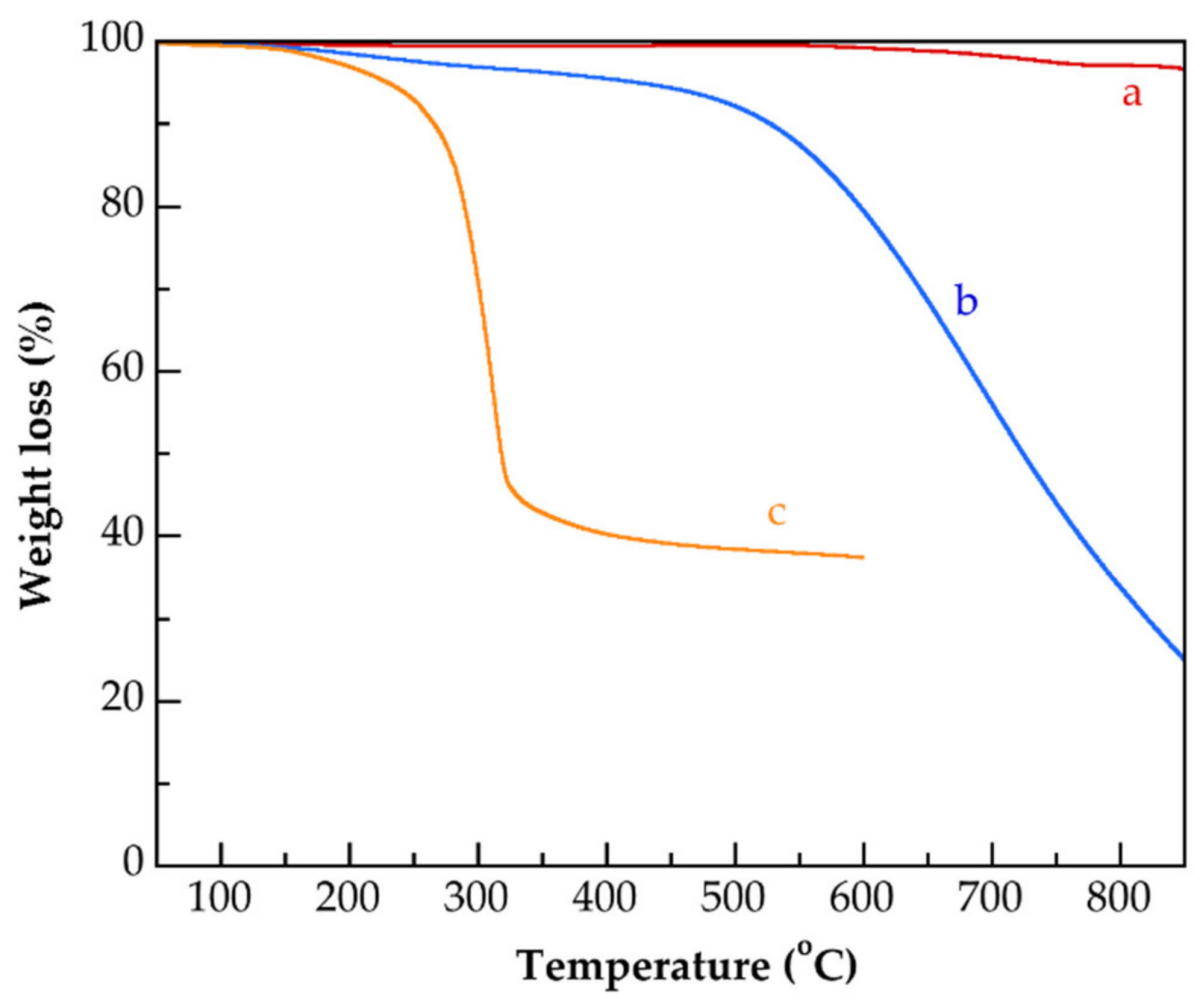
T_g_ thermograms of CNTspur (**a**), CNTsoxid (**b**), and G3.0-CNTs-PAMAM-NH_2_ (**c**).

**Figure 9:**
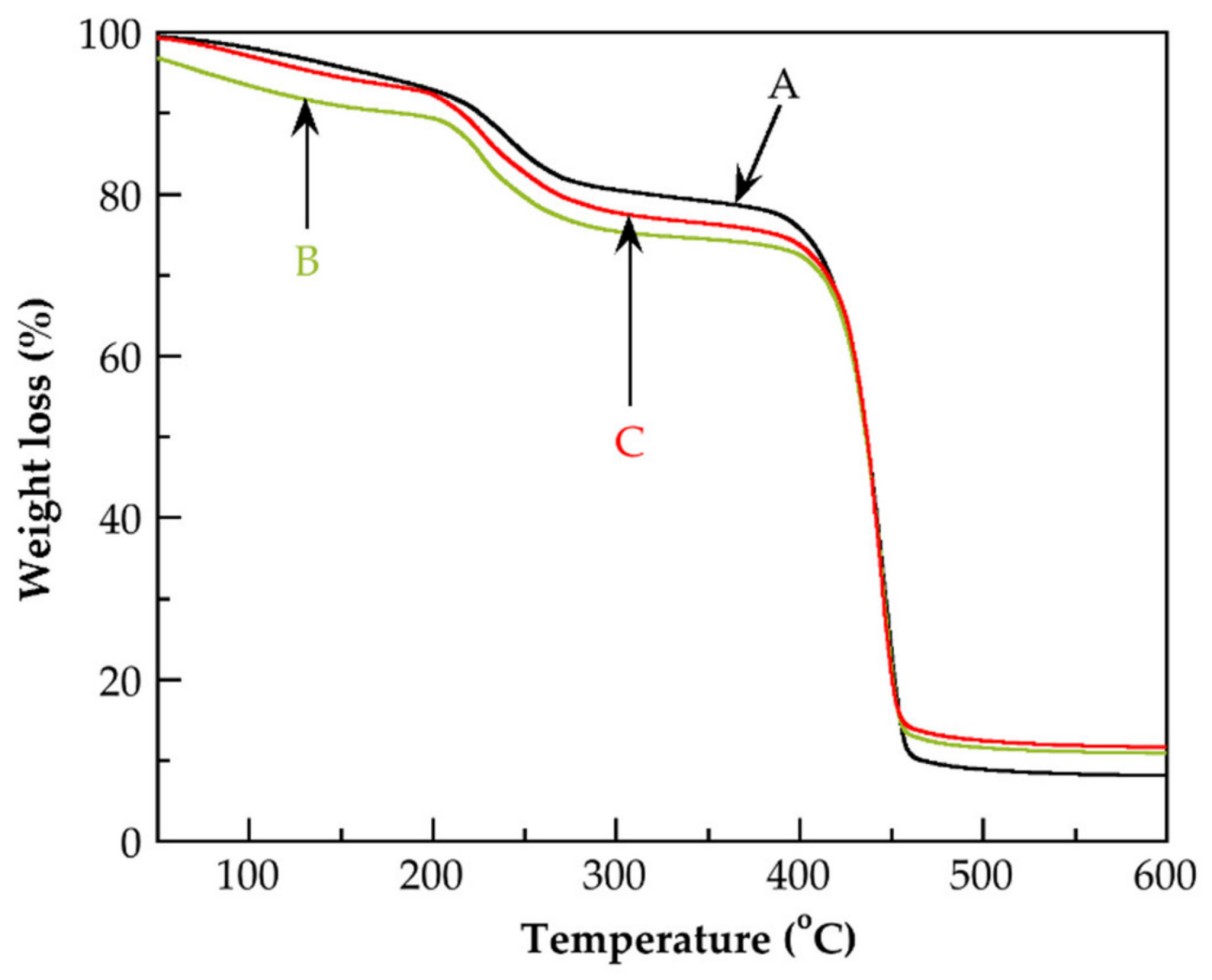
TG thermograms of PMAA 3 (**A**), PMAA-G_3.0_-CNTs 5 (**B**), and PMAA-G_3.0_-CNTs 6 (**C**).

**Figure 10:**
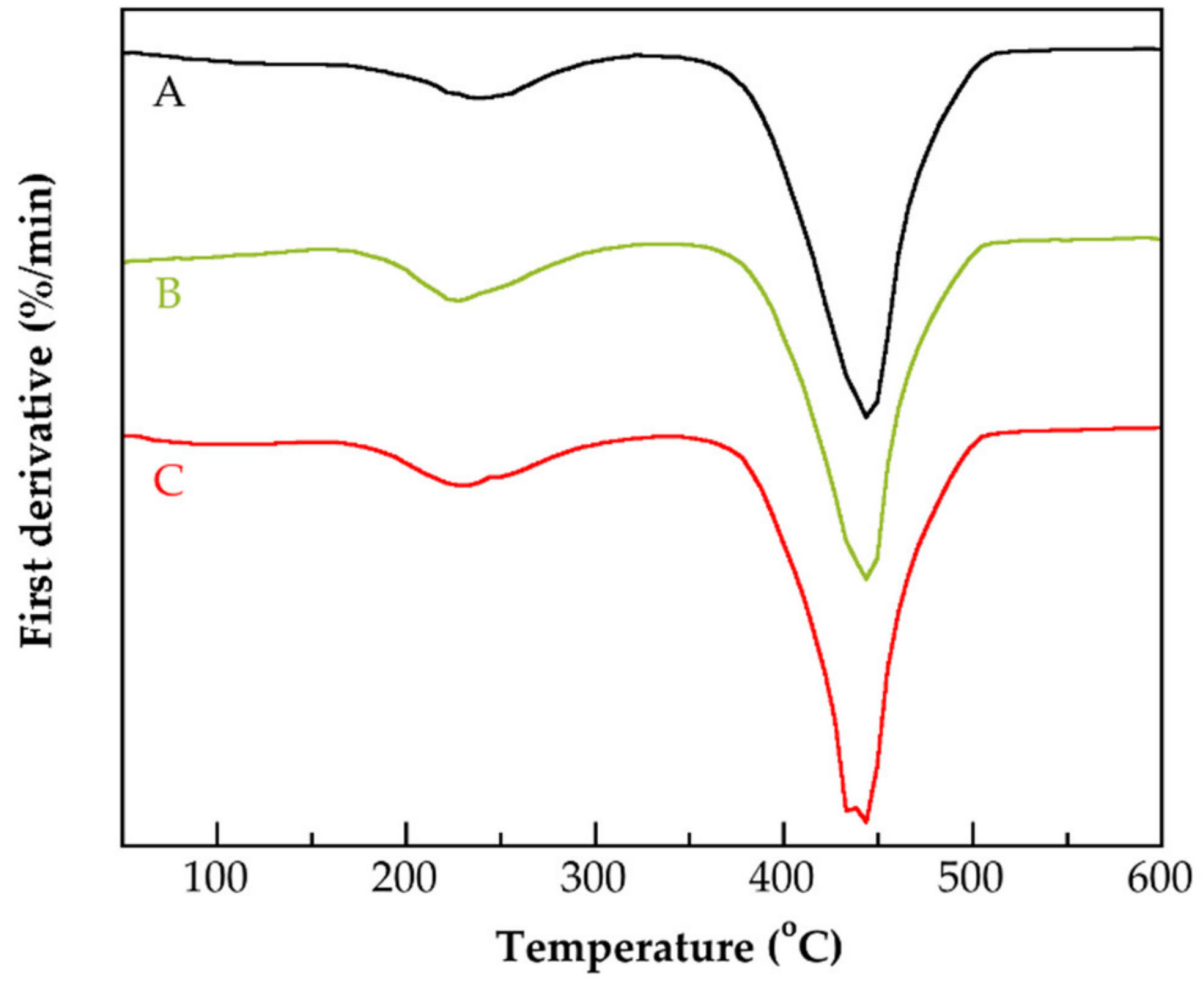
DTG thermograms of PMAA 3 (**A**), PMAA-G_3.0_-CNTs 5 (**B**), and PMAA-G_3.0_-CNTs 6 (**C**).

In order to show more evidence with respect to the success of the grafting reaction, an analysis using the TGA technique was performed. Figure 8 shows thermogravimetric (T_g_) curves of CNTspur, CNTsoxid, and G3.0-CNTs-PAMAM-NH_2_. The TG curve of CNTspur (Figure 8a) practically does not show a weight loss in the range from 50 to 850 °C, only a small decrease of ca. 3 wt.% was detected at 850 °C. This thermal behavior confirms the well-known thermal resistance of the CNTs. When CNTspur were functionalized, the thermal resistance decreased. For CNTsoxid (Figure 8b), a slight weight loss begins at ca. 200 °C, while at ca. 470 °C, a drastic weight loss starts, which continues until 850 °C, which is associated with the degradation of carboxyl, hydroxyl, carbonyl groups, and carbon structure of CNTsoxid. For G3.0-CNTs-PAMAM-NH_2_ (Figure 8c), the thermal degradation starts at ca. 160 °C. Further, in the range from 225 °C to ca. 380 °C, the main weight loss takes place. Obviously, in this temperature range, PAMAM dendrimer chains were degraded thermally, as can be deduced when comparing this result with other results reported elsewhere. In this sense, Ozturk et al. reported that for a PAMAM of the fourth generation, a greater fraction of PAMAM decomposition occurs below 300 °C, while the complete degradation was reached above 550 °C [66]. Moreover, in the G3.0-CNTs-PAMAM-NH_2_ TG curve, a residual weight of ca. 38 wt.% remains at 600 °C was detected, and that can be associated with CNTs that keep their structure. Figure 9 shows T_g_ curves, while Figure 10 depicts the first derivative thermogravimetric curves (DTA) of PMAA 3, PMAA-G_3.0_-CNTs 5, and PMAA-G_3.0_-CNTs 6. All mentioned materials were prepared at the same monomer-feeding rate (0.2 g/min). A total of two clear weight losses can be observed in these TG-thermograms: (i) the first is observed from ca. 190 °C to 270 °C, (ii) the second occurs from ca. 380 °C to 470 °C. From DTA curves were calculated the minimum temperature of the first transition resulting in 240 °C for PMAA 3, 227 °C for PMAA-G_3.0_-CNTs 5, and 230 °C for PMAA-G_3.0_-CNTs 6. In a similar way, from DTA curves was determined the minimum temperature of the second transition, which was detected at the same temperature (443 °C) for PMAA 3, PMAA-G_3.0_-CNTs 5, and PMAA-G_3.0_-CNTs 6. Ho et al. studied the thermal degradation of pure PMAA by thermogravimetric analysis following isothermal experiments [67]. They reported a two-steps thermal mechanism of degradation as a consequence of heating PMAA, that transforms this polymer into poly(methacrylic anhydride) (PMAN), which contains six-membered glutaric anhydride-type rings. The first decomposition region is due to anhydride group formation, which causes the conversion of PMAA to PMAN. The second decomposition was produced by the fragmentation of anhydride rings in the PMAN structure. Schild confirms the existence of the mentioned two-steps thermal degradation when the degradation test was performed following a temperature ramp [68]. From the DTA results, it is evident that in the nanocomposites, the first decomposition is shifted to lower temperatures. This fact strongly suggests that some carboxyl groups of their polymeric matrix previously reacted chemically. Because if low amounts of carboxyl groups form part of the nanocomposites’ structure, lower temperature are needed to produce the anhydride functionality. As was analyzed by XPS, some carboxyl groups of the PMAA chains could have reacted with –NH_2_ groups of the G_3.0_-CNTs-PAMAM-NH_2_. The TGA results confirm that amidation reaction effectively occurred. The small amount of G3.0-CNTs-PAMAM-NH_2_ used to prepare the studied nanocomposites implies that the thermal degradation of mentioned nano-filler does not prevail over the ones of the polymeric matrix.

### 3.4. Thermal Characterization

**Figure 11:**
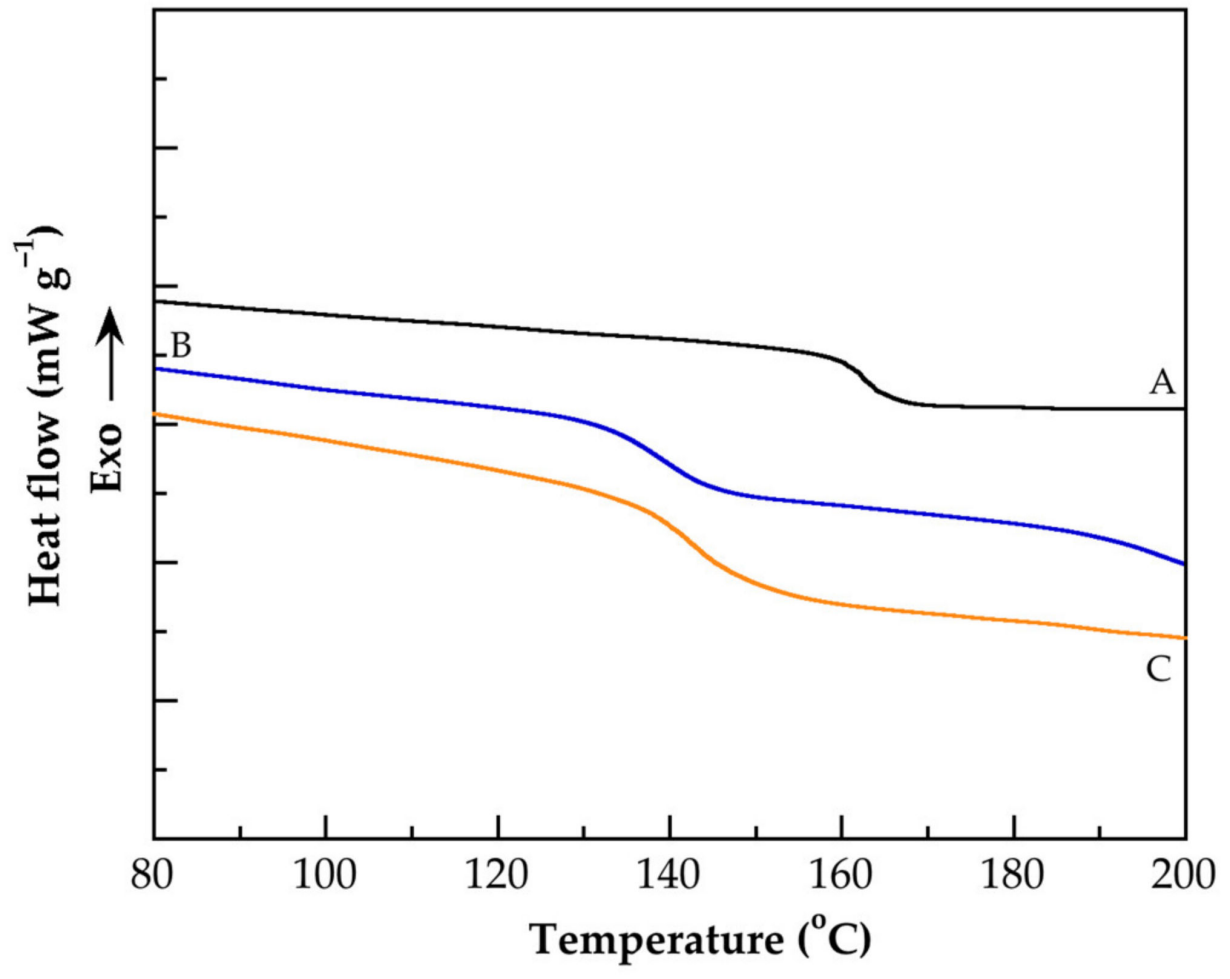
DSC thermograms of PMAA 2 (**A**), and of their nanocomposites: PMAA-G_3.0_-CNTs 3 (**B**) and PMAA-G_3.0_-CNTs 4 (**C**).

**Figure 12:**
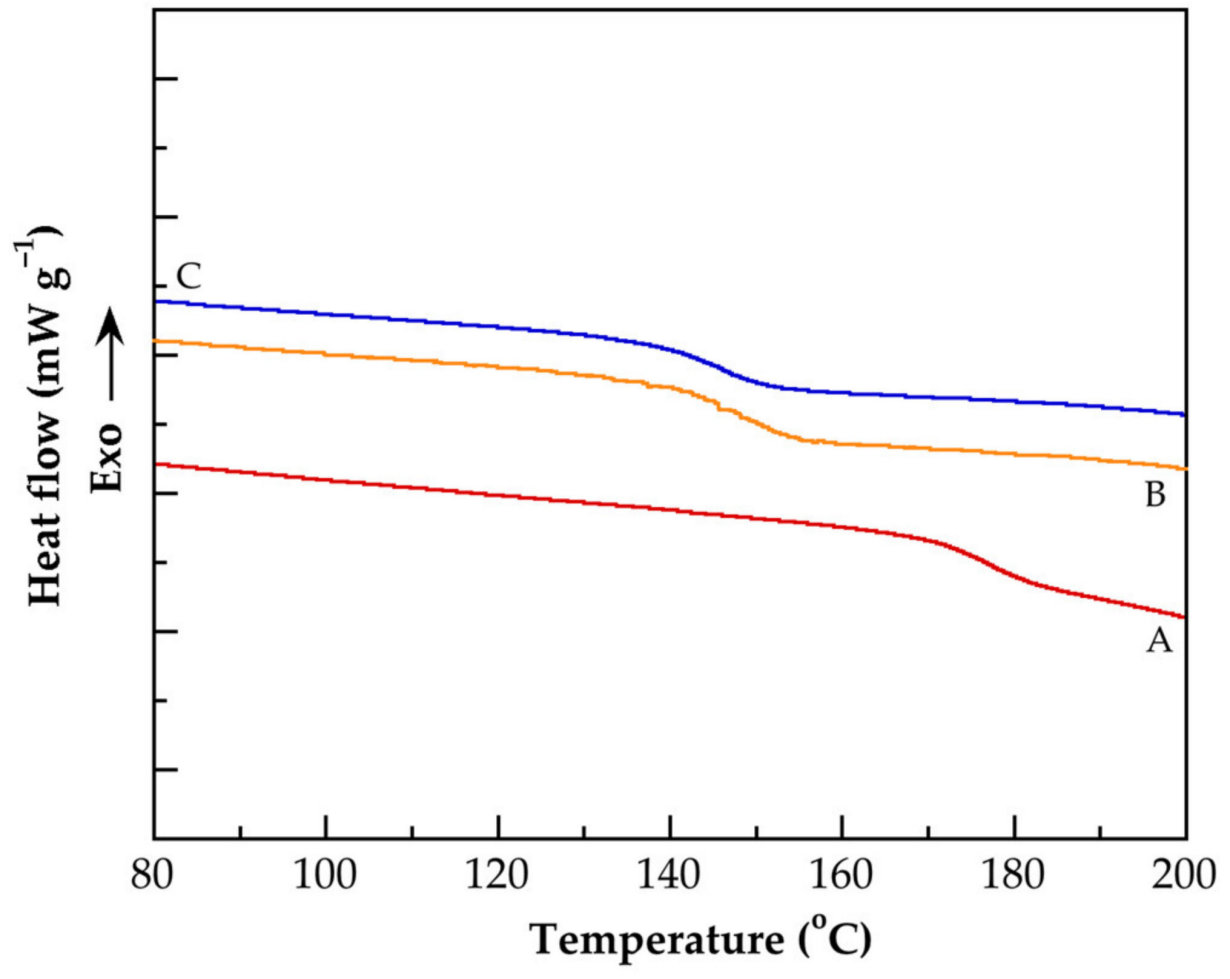
DSC thermograms of PMAA 5 (**A**), and of their nanocomposites: PMAA-G_3.0_-CNTs 10 (**B**), and PMAA-G_3.0_-CNTs 9 (**C**).

Figure 11 depicts DSC thermograms of PMAA 2, and of the nanocomposites PMAA-G_3.0_-CNTs 3 and PMAA-G_3.0_-CNTs 4, while Figure 12 shows DSC thermograms of PMAA 5, and of the nanocomposites PMAA-G_3.0_-CNTs 9 and PMAA-G_3.0_-CNTs 10. PMAA is an amorphous polymer and the only thermal transition that it experiences is the glass relaxation. The same thermal behavior was detected in the DSC thermograms of the prepared nanocomposites. Table 3 lists the values of the glass transition temperatures (T_g_s) of the pure PMAAs and of all nanocomposites prepared. Taking into consideration that the same monomer-feeding rate was used to prepare all these materials, it is evident that the T_g_ of pure PMAAs is higher than those of their nanocomposites. Specifically, T_g_ of PMAA 2 is higher than of PMAA-G_3.0_-CNTs 3, or of the PMAA-G_3.0_-CNTs 4. These nanocomposites and their pure polymeric matrix were prepared at a same monomer-feeding rate (0.1 g/min). In a similar way, the T_g_ of PMAA 5 was also detected at higher temperatures than the T_g_ of PMAA-G_3.0_-CNTs 9, or of the PMAA-G_3.0_-CNTs 10. A monomer-feeding rate of 0.3 g/min was used to prepare these last materials. A similar behavior was observed for the nanocomposites and their pure polymeric matrix prepared at monomer-feeding rate of 0.2 g/min. For the nanocomposites prepared at a monomer-starved condition, the more remarkable depression among values of T_g_s was detected comparing the T_g_ of PMAA 2 and the one of PMAA-G_3.0_-CNTs 3 (prepared with 0.5 wt.% of G_3.0_-CNTs-PAMAM-NH_2_ and 8 wt.% of MAA), which was 23 °C, while when the mentioned condition was not reached, a similar depression was detected, of 27 °C between the T_g_ of PMAA 5 and the T_g_ of the PMAA-G_3.0_-CNTs 9 (prepared with 0.5 wt.% of G_3.0_-CNTs-PAMAM-NH_2_ and 4 wt.% of MAA). These results suggest that neither the monomer-starved condition nor the amount of MAA used in the synthesis determine the decrease in the value of T_g_. On the contrary, a low amount of the functionalized nanofiller (0.5 wt.%), which favors a good dispersion, seems to induce the thermal behavior observed. In other works, the decrease in the value of the T_g_ for other studied nanocomposites has also been detected. Babal et al. reported a depression of 2 °C in the T_g_ of nanocomposites prepared with functionalized MWCNTs and polycarbonate (PC) as a polymer matrix, with respect to pure PC [69]. They consider that a good dispersion of functionalized MWCNTs, and formation of high amounts of thin film of nano-scale confined polymer between the MWCNTs is the cause of T_g_ depression of their studied nanocomposites. In a similar way, Khare et al., using an atomistic molecular simulation, studied the effect of dispersion of CNTs on the T_g_ of cross-linked epoxy–CNTs nanocomposites. They found that the nanocomposites studied containing dispersed CNTs show a depression in the T_g_ by 66 °C as compared to the neat, cross-linked epoxy, but if CNTs formed aggregated domains, no T_g_ depression was observed [70]. Additionally, the free-radical scavenging capacity of the CNTs is another factor that favors the decreasing of the nanocomposites’ T_g_, because the presence of free radicals on the surface of CNTs induces the appearance of species that can induce the decomposition of polymer chains [71], thereby promoting the reduction of molecular weight [72]. Our results confirm the key role of CNTs’ dispersion and their scavenger capacity on the nanocomposites’ T_g_ depression. However, there is no evidence that the monomer-starved condition induces the creation of a more ordered structure in the prepared nanocomposites, which could have favored a homogeneous dispersion of the functionalized CNTs. It is important to note that for the pure PMAA synthesized, an increment of the T_g_ values as the monomer feed rate increased was observed (Table 3). As was reported previously, the monomer feed rate influences the T_g_ of polymers synthesized via *SHP*, which is associated with tacticity formation in methacrylic polymers [38]. The increment on the T_g_ of PMAA observed suggests the formation of more intense intermolecular interactions between PMAA chains. This result indicates that the monomer feed rate used has an influence on morphology of PMAAs synthesized. In fact, the highest T_g_ detected (179 °C at R_a_ = 0.3 g/min) is nearer to T_g_ of PMAA (228 °C) reported elsewhere [73].

### 3.5. Release of Hydrocortisone from PMAA and of Their Nanocomposites

**Figure 13:**
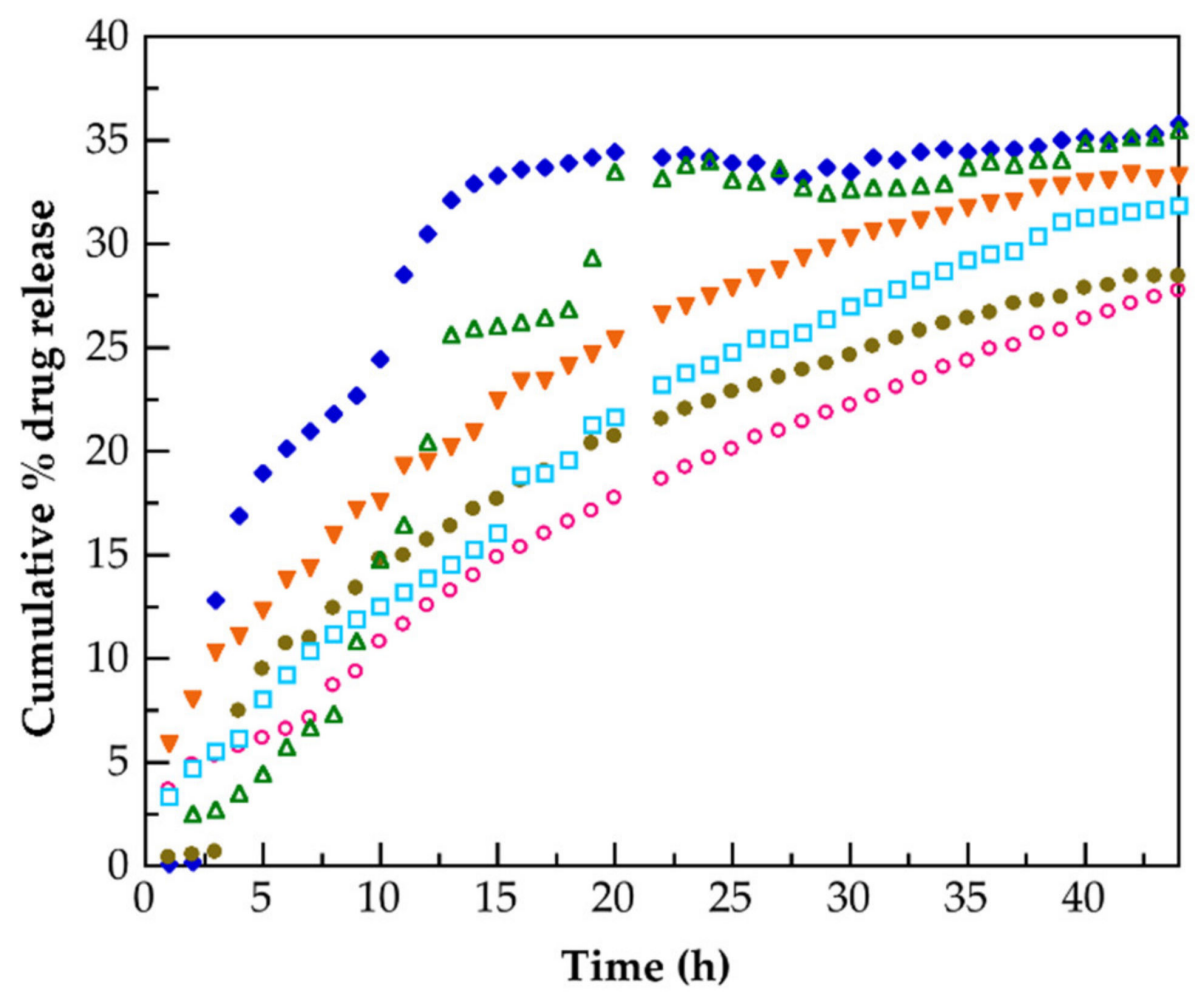
Hydrocortisone release profiles for PMAA 1 (◯), PMAA 2 (●), PMAA-G_3.0_-CNTs 1 (▼), PMAA-G_3.0_-CNTs 2 (◆), PMAA-G_3.0_-CNTs 3 (☐), and PMAA-G_3.0_-CNTs 4 (△).

Figure 13 presents the hydrocortisone in vitro release profiles for PMAA 1, PMAA 2, PMAA-G_3.0_-CNTs 1, PMAA-G_3.0_-CNTs 2, PMAA-G_3.0_-CNTs 3, and PMAA-G_3.0_-CNTs 4. For these nanocomposites, the monomer-starved condition was reached during their preparation. In this figure, it can be observed that the profile release of the nanocomposites is higher than of their pure polymeric matrices (PMAA 1 and PMAA 2). The higher hydrocortisone release ability was detected in PMAA-G_3.0_-CNTs 2 and PMAA-G_3.0_-CNTs 4 (ca. 25.5 % at 44 h). Both were prepared with the same content of G_3.0_-CNTs (1 wt.%). These results indicated that the presence and the content of CNTs in the nanocomposites play a crucial role in the hydrocortisone release due to its capacity to act as drug reservoirs, which has been reported elsewhere [13]. Moreover, the PAMAM dendrimer chains attached to these CNTs favors the retention of drugs and the prior release [32] at the pH used in this work.

The five models used to fit the experimental release data of hydrocortisone were: zero-order (3), first-order (4), Higuchi (5), Korsmeyer–Peppas (6), and Hixson–Crowell (7):

$$C=K_{0}t$$

where the cumulative amount of drug released versus time is plotted. $K_{0}$ is the zero-order rate constant (obtained from the slope of a straight line of the concentration (C) of drug vs. time (*t*)) and the intercept is the origin of the axes [74].

$$Log\;C=Log\;C_{0}-\frac{K_{I}\cdot t}{2.303}$$

in which, $K_{1}$ is the first-order constant and $C_{0}$ is the initial concentration of drug, and *t* is the time [75].

$$Q=K_{H}\cdot t^{\frac{1}{2}}$$

where $K_{H}$ is the rate constant of the Higuchi model and is associated with the design variables of the release system, *Q* is the amount of drug released, and *t* is the time [76].

$$\frac{M_{t}}{M_{\infty }}=K_{KP}\cdot t^{n}$$

here, $K_{KP}$ is the kinetic rate constant of Korsmeyer–Peppas’ model $M_{t}/M_{\infty }\;$ is fraction of drug released at time *t*, and *n* is the release exponent, which is used to characterize the release mechanism from polymeric matrices with different geometries. This model was developed specifically to study release of drugs from a polymeric matrix [77].

$$\sqrt[{3}]{Q_{0}}-\sqrt[{3}]{Q_{t}}=K_{H}\cdot t$$

where $K_{HC\;}$is the rate constant of Hixson–Crowell rate equation, $Q_{0}$ is the initial amount of drug in the tablet, and $Q_{t}$ is the amount of drug released in the time *t* [78].

**Figure 14:**
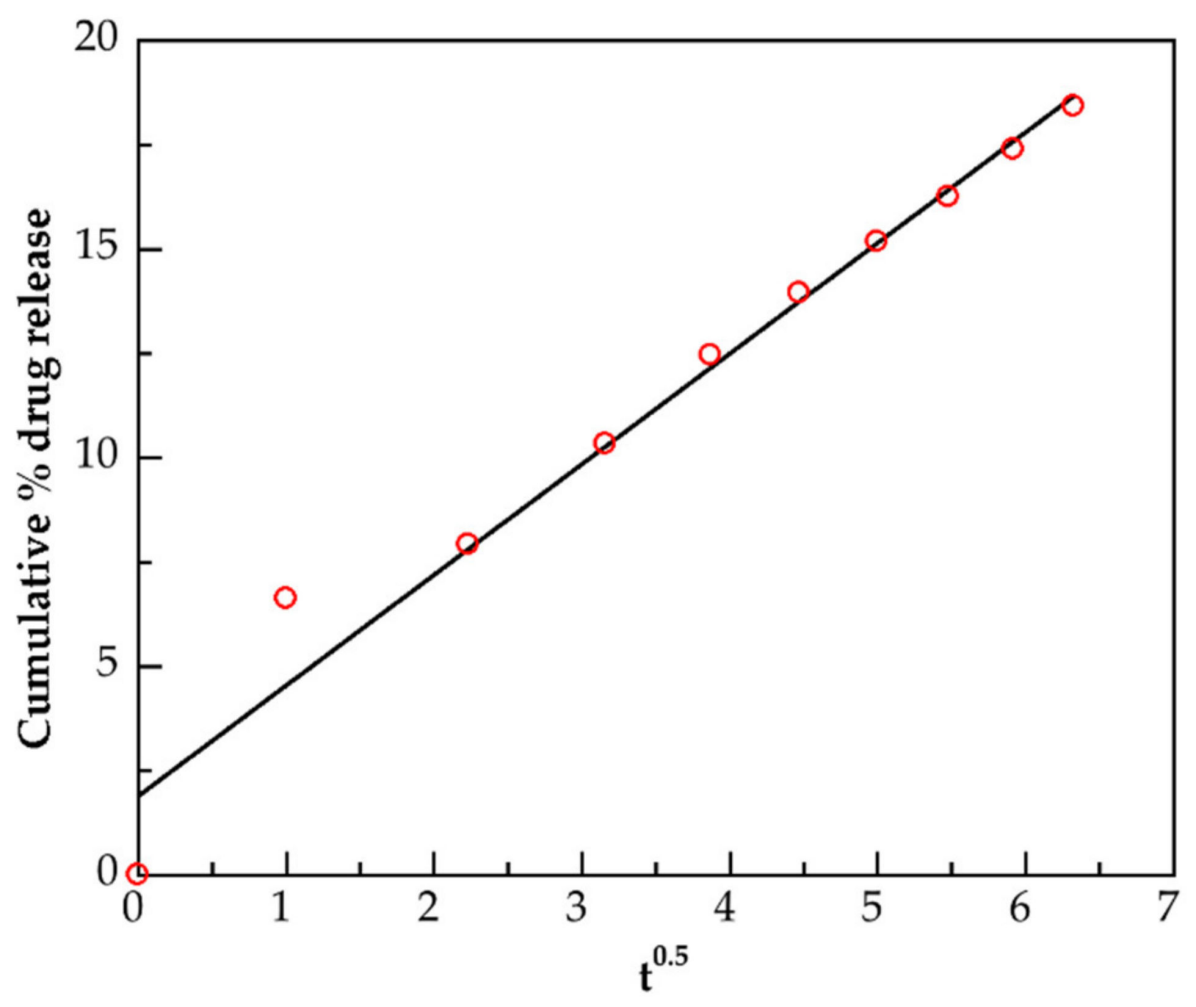
Fit of experimental data of hydrocortisone release for PMAA 1 (◯) with Higuchi model (continuous line).

**Figure 15:**
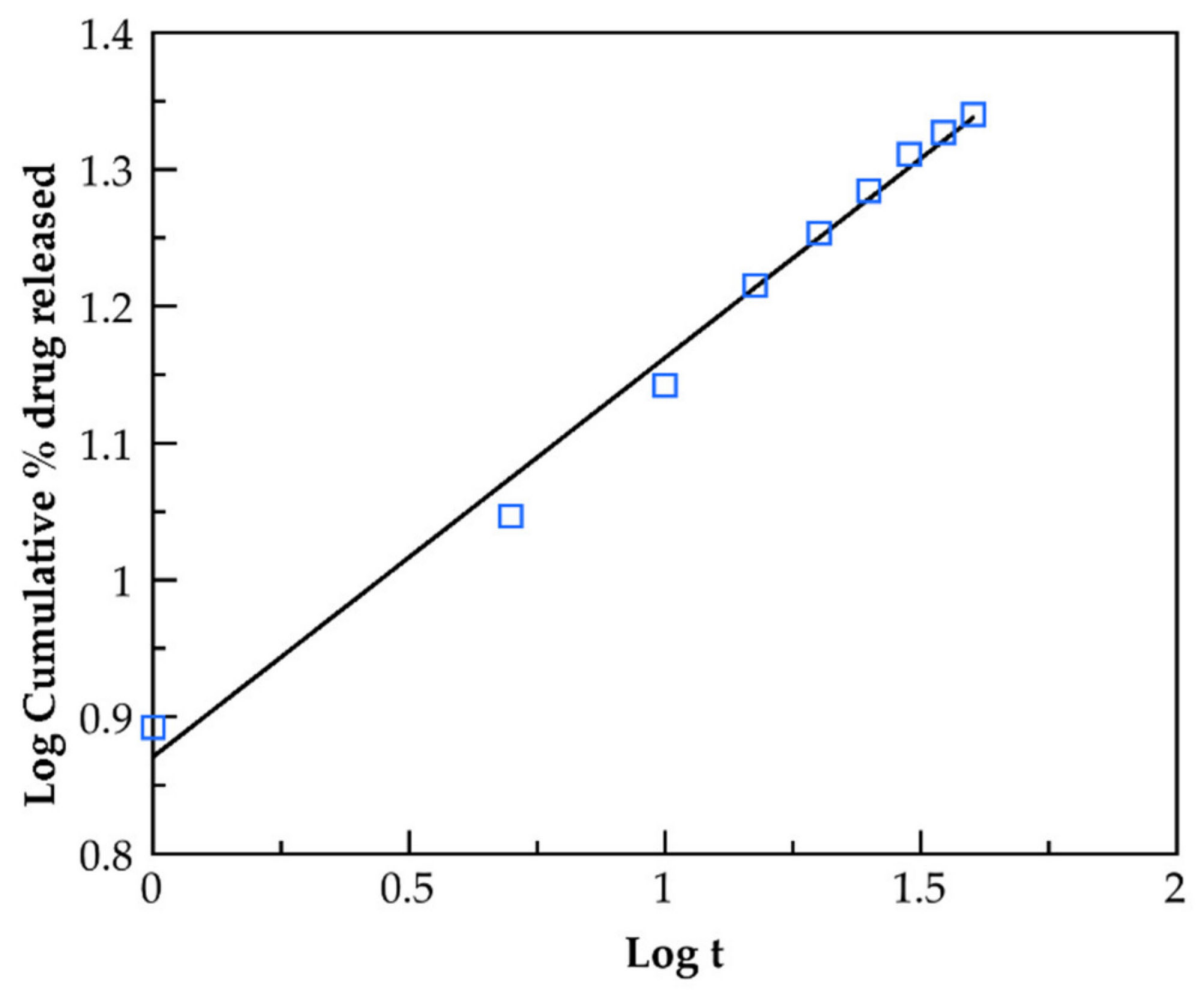
Fit of experimental data of hydrocortisone release for PMAA-G_3.0_-CNTs 1 (☐) with Korsmeyer–Peppas model (solid line).

To fit the experimental data of the hydrocortisone release to mentioned models, the program Origen Pro 8 was used. Table 4 lists the values of the kinetic rate constants of the drug release for the five mentioned models, as well as the R^2^ values founded from the best fit. It is evident that zero-order, first-order, and Hixson–Crowell models can be ruled out due to the low R^2^ values calculated. These results indicate that for the hydrocortisone release rate from studied materials, the concentration of this drug does not play a relevant influence, as can be evaluated with zero-order or first-order models. A similar assessment was performed with respect to the role of changes in the surface area of the solid used as a carrier of the drug, which can be evaluated with the Hixson–Crowell model. Comparatively, a better fix was observed for the Korsmeyer–Peppas and Higuchi models. Figure 14 and Figure 15 how the fit for PMAA 1 with Higuchi model, and for PMAA-G_3.0_-CNTs 1 with the Korsmeyer–Peppas model, respectively. The Higuchi model describes the release of drugs based on Fickian diffusion. This model was obtained considering homogeneous (as the pure PMAA 1 and PMAA 2) or heterogeneous (as the nanocomposites prepared) release systems. In a similar way, the Korsmeyer–Peppas model is useful when more than one process occurs during drug release phenomenon. In this work, the release exponent (*n*) was determined using the portion release curve where $\frac{M_{t}}{M_{\infty }}$ < 0.60. Fickian diffusion is detected when *n* is 0.50 for a planar (thin films) matrix, while *n* is 0.43 for a matrix of a sphere-shape, respectively. Our results show that *n* for both PMAA-G_3.0_-CNTs 2 and PMAA-G_3.0_-CNTs 4, the nanocomposites is 0.41. Therefore, for both mentioned nanocomposites, prepared with 1 wt.% of G_3.0_-CNTs, these results strongly suggest that Fickian diffusion drives the hydrocortisone release. For PMAA 1, PMAA 2, and other nanocomposites studied, *n* exponent is more minor than 0.43, and is beyond the limits of the Korsmeyer–Peppas model. Nevertheless, as was reported in other works [79], also for these materials, diffusion would control the hydrocortisone release mechanism. Due to the fact that diffusion is a secure, efficient, and simple way to release several drugs with control over a short distance [80], our results could be of practical use.

## 4. Conclusions

Our results show that nanocomposites of PMAA grafted onto CNTs functionalized with PAMAM dendrimer were prepared successfully by *SHP*, in order to release hydrocortisone in a more effective way than neat PMAA. The monomer-starved condition was detected for the nanocomposites prepared with R_a_ = 0.1 g/min, but this condition was not achieved for the pure PMAA matrix prepared at the same R_a_. To develop this behavior, the free-radical scavenging capacity of the CNTs has a positive influence. The monomer-starved condition was lost for all the prepared materials as R_a_ increased. In the DSC thermogram, results show that there is not evidence that nanocomposites prepared under a monomer-starved condition contain ordered structures. The grafting of PMAA chains onto functionalized CNTs with PAMAM dendrimer was confirmed by XPS spectroscopy. TGA measurements confirm that the amidation reaction occurs, probing that PMAA chains are grafted to the nanofiller (G3.0-CNTs-PAMAM-NH_2_). A high dispersion of functionalized CNTs induced for a lower amount of G3.0-CNTs-PAMAM-NH2 (0.5 wt.%) causes the T_g_ (determined by DSC tests) of the prepared nanocomposites to decrease markedly with respect to the T_g_ of the pure PMAA matrix. Nanocomposites prepared with a content of 1 wt.% of G_3.0_-CNTs-PAMAM-NH_2_ show a better ability to release hydrocortisone, indicating that both the presence and the content of the nano-filler have a crucial role to induce an efficient hydrocortisone delivery. This is a consequence of the ability of the G_3.0_-CNTs-PAMAM-NH_2_ to act as a drug reservoir. The hydrocortisone release of the nanocomposites prepared under a monomer-starved condition was fitted with Higuchi and Korsmeyer–Peppas models successfully.

## Figures and Tables

**Table 1:** Legends that identify the synthesized PMAA and their nanocomposites as a function of the formulation used and the rate of monomer feed. The content of G_3.0_-CNTs-PAMAM-NH_2_ was calculated with respect to the mass of the MAA used.

Identification Name	Content of MAA ^1^ (wt.%)	Content of G_3.0_-CNTs-PAMAM-NH_2_ ^2^ (wt.%)	Rate of Addition of MAAor the Mixture of MAA/G_3.0_-CNTs-PAMAM-NH_2_ (g/min)
PMAA 1	4	0	0.1
PMAA 2	8	0	0.1
PMAA 3	4	0	0.2
PMAA 4	8	0	0.2
PMAA 5	4	0	0.3
PMAA 6	8	0	0.3
PMAA-G_3.0_-CNTs 1	4	0.5	0.1
PMAA-G_3.0_-CNTs 2	4	1.0	0.1
PMAA-G_3.0_-CNTs 3	8	0.5	0.1
PMAA-G_3.0_-CNTs 4	8	1.0	0.1
PMAA-G_3.0_-CNTs 5	4	0.5	0.2
PMAA-G_3.0_-CNTs 6	4	1.0	0.2
PMAA-G_3.0_-CNTs 7	8	0.5	0.2
PMAA-G_3.0_-CNTs 8	8	1.0	0.2
PMAA-G_3.0_-CNTs 9	4	0.5	0.3
PMAA-G_3.0_-CNTs 10	4	1.0	0.3
PMAA-G_3.0_-CNTs 11	8	0.5	0.3
PMAA-G_3.0_-CNTs 12	8	1.0	0.3

1: methacrylic acid; 2: third-generation poly(amidoamine) attached to functionalized CNTs.

**Table 2:** Wavelengths of the representative bands and chemical group that cause them for each type of functionalized CNTs whose chemical structure was presented in the Scheme 1.

Name of Functionalized CNTs	Wavelength (cm^−1^)	Chemical Group
CNTs_oxid_	3433	O–H stretching
CNTs_OxCl_	1668 808 737	di-carbonyl stretching C–Cl stretching C–Cl stretching
G_0.0_-CNTs-PAMAM-NH_2_	1663 1123	C=O stretching C–N stretching
G_0.5_-CNTs-PAMAM-OCH_3_	1699 1432	C=O stretching C–H symmetric deformation
G_3.0_-CNTs-PAMAM-NH_2_	1641 1112	C=O stretching C–N stretching
Mixture of G3.0-CNTs-PAMAM-NH2 and MAA	1637 3431	N–H bending N–H stretching

**Table 3:** Values of the glass transition temperature of the synthesized PMAAs and of their nanocomposites.

Identification Name	T_g_ (°C)
PMAA 1	155
PMAA 2	163
PMAA 3	163
PMAA 4	165
PMAA 5	179
PMAA 6	159
PMAA-G_3.0_-CNTs 1	148
PMAA-G_3.0_-CNTs 2	149
PMAA-G_3.0_-CNTs 3	140
PMAA-G_3.0_-CNTs 4	142
PMAA-G_3.0_-CNTs 5	151
PMAA-G_3.0_-CNTs 6	157
PMAA-G_3.0_-CNTs 7	152
PMAA-G_3.0_-CNTs 8	150
PMAA-G_3.0_-CNTs 9	155
PMAA-G_3.0_-CNTs 10	149
PMAA-G_3.0_-CNTs 11	153
PMAA-G_3.0_-CNTs 12	149

**Table 4:** Kinetic parameters for hydrocortisone release from tablets of PMAA 1, PMAA 2, and of their nanocomposites obtained with zero-order, first-order, Higuchi, Korsmeyer–Peppas, and Hixson–Crowell models.

Tested Materials	Models										
	Zero-Order		First-Order		Higuchi		Korsmeyer–Peppas			Hixson–Crowell	
	R^2^	*K_0_* (h^−1^)	R^2^	*K_I_* (h^−1^)	R^2^	*K_H_* (h^–1/2^)	R^2^	*n*	*K_KP_* (h^–n^)	R^2^	*K_HC_* (h^–1/3^)
PMAA 1	0.85	0.37	0.91	0.011	0.97	2.65	0.93	0.29	5.80	0.43	0.039
PMAA-G_3.0_-CNTs 1	0.79	0.44	0.82	0.010	0.96	3.22	0.99	0.29	7.43	0.40	0.040
PMAA-G_3.0_-CNTs 2	0.61	0.47	0.43	0.012	0.85	3.66	0.88	0.41	6.01	0.48	0.021
PMAA 2	0.80	0.40	0.71	0.012	0.97	2.91	0.99	0.36	5.15	0.77	0.021
PMAA-G_3.0_-CNTs 3	0.88	0.45	0.89	0.012	0.98	3.13	0.96	0.33	5.78	0.47	0.042
PMAA-G_3.0_-CNTs 4	0.78	0.53	0.71	0.014	0.93	3.81	0.91	0.41	5.37	0.48	0.046

## Data Availability

The data presented in this study are available on request from the corresponding author.
